# A deep phenotyping approach to assess the association of handedness, early life factors and mental health

**DOI:** 10.1038/s41598-023-42563-7

**Published:** 2023-09-15

**Authors:** Lena Sophie Pfeifer, Judith Schmitz, Maike Schwalvenberg, Onur Güntürkün, Sebastian Ocklenburg

**Affiliations:** 1 Cognitive Psychology, Institute of Cognitive Neuroscience, Faculty of Psychology, Ruhr University Bochum, Bochum, Germany; 2 Biological Personality Psychology, Georg-August-University Goettingen, Göttingen, Germany; 3 Biopsychology, Institute of Cognitive Neuroscience, Faculty of Psychology, Ruhr University Bochum, Bochum, Germany; 4 Department of Psychology, Medical School Hamburg, Hamburg, Germany; 5 Institute for Cognitive and Affective Neuroscience, Medical School Hamburg, Hamburg, Germany

**Keywords:** Genetics, Psychology

## Abstract

The development of handedness and other form of functional asymmetries is not yet understood in its critical determinants. Early life factors (e.g., birth weight, birth order) have been discussed to contribute to individual manifestations of functional asymmetries. However, large-scale data such as the UK Biobank suggest that the variance in handedness that is explained by early life factors is minimal. Additionally, atypical handedness has been linked to clinical outcomes such as neurodevelopmental and psychiatric disorders. Against the background of this triad, the current study investigated associations between different forms of functional asymmetries and (a) early life factors as well as (b) clinical outcomes. Functional asymmetries were determined by means of a deep phenotyping approach which notably extends previous work. In our final sample of *N* = 598 healthy participants, the different variables were tested for associations by means of linear regression models and group comparisons (i.e., ANOVAs and Chi-squared tests). Confirming previous findings from larger cohorts with shallow phenotyping, we found that birth factors do not explain a substantial amount of variance in functional asymmetries. Likewise, functional asymmetries did not seem to have comprehensive predictive power concerning clinical outcomes in our healthy participants. Future studies may further investigate postulated relations in healthy and clinical samples while acknowledging deep phenotyping of laterality.

## Introduction

Functional asymmetries are widespread across species^1,2^ and can be found in simple motor tasks^2^, complex socio-behavioral patterns^3,4^, and in cognitive information processing^5,6^. For humans, the most obvious form of lateralization is handedness. Importantly, human handedness is not only asymmetric on an individual level but also on a population level. More precisely, a recent meta-analysis estimated that 10.6% of the population is left-handed^7^. Despite such clear evidence on the phenotypic level, it is still largely unknown in how far genetic and environmental factors contribute to the development of individual handedness and other forms of functional asymmetries^8^. Similarly, it is an open question how far the ontogenesis of a lateralized brain may overlap with developmental pathways of psychopathology. In this regard, several neurodevelopmental and psychiatric disorders have been associated with atypical lateralization^9^. Unraveling causal mechanisms in the development of structural and functional asymmetries may hence have clinical relevance.

Early (mono-)genetic theories on the development of handedness^10,11^ have been refuted as being too simplistic^12^. Likewise, candidate genes initially found to show associations with handedness could often not be replicated^13^. Along these lines, twin studies confirm that genetic factors explain about a quarter of the variance in human handedness^14,15^. Twin studies estimate the additive genetic heritability of a trait by comparing phenotypic concordance between monozygotic and dizygotic twins. Genome-wide association studies (GWAS) take a molecular approach in that millions of single nucleotide polymorphisms (SNPs) are tested for an association with the phenotype of interest. The largest GWAS on handedness so far (*N* = 1,766,671) suggested common SNPs account for only 3.45–5.9% of the variance^16^. Interestingly, significant loci were located in genes playing a role in microtubule formation and regulation. Microtubules are protein complexes that form the cellular cytoskeleton and support neurogenesis, neuronal migration and axonal transport^17^. Noteworthy, mutations of microtubule-related genetic variants have also been linked to neurodevelopmental^18,19^ and neurodegenerative disorders^19^. Handedness as considered on a population level probably disposes on a strong genetic basis which is clearly evident in the overwhelming bias towards right-handedness. That is, right-handedness likely reflects a common manifestation of brain asymmetries that already develop in the majority of fetuses^20,21^. Still, in summary, existing literature points towards a small role of genetic factors in the development of individual handedness deviating from this population-level bias (e.g., left-handedness).

For a long time, phenotypic variation has been assumed to arise from genetic or non-genetic sources while the latter component was defined as being environmental^22^. Since Medland et al.^14^ found no evidence for shared environmental variance to play a role in handedness development, it has often been concluded that non-shared environmental variance must account for the remaining proportion of unexplained variance in handedness^23^. For example, de Kovel et al.^24^ investigated large-scale data from the UK Biobank for an association between early life factors (e.g., birth weight, maternal smoking) and adult left-handedness. Indeed, adult left-handedness was shown to correlate with birth year and birth location—an effect that de Kovel et al.^24^ attributed to cultural artifacts. This is in line with studies indicating handedness to reflect interactions between genetic factors and cultural influences in terms of parenting, teaching, and implicit model learning^25,26^. Moreover, in the study by de Kovel et al.^24^, birthweight, multiple status, season of birth, maternal breastfeeding, and the participant’s sex were associated with left-handedness. However, even the combined predictive power of these factors was only marginal. McManus^23^ hence put forward that virtually none of the 75% of variance remaining unexplained from above-mentioned twin studies can be explained by environmental factors. Therefore, it has been argued that unexplained variance in handedness or other forms of functional asymmetries may not be environmental in the stricter sense but includes developmental noise, randomness^22,23^ and measurement error^27^. Graham^22^ continues that some portion of randomness may not be solved by applying stochastic rules to the behavior of involved biological agents but that other laws of dynamical systems such as deterministic chaos may play a role. Similarly, de Kovel et al.^24^ suggested that probabilistic randomness in terms of a ‘random model of early embryonic development’ might also contribute to the ontogenesis of individual left-handedness. That is, gene expression might underlie some sort of gradient which may be lateralized across embryonic brains on average^28,29^, but also includes symmetry or a reversal of asymmetry on the individual level.

Epigenetic regulation has been suggested as a mechanism linking environmental factors and phenotypic outcomes in that environmental factors can modulate gene expression without modifying the actual nucleotide sequence. With respect to handedness, Schmitz et al.^30^ reviewed the evidence for environmental factors previously associated with handedness (e.g., season of birth, intrauterine environment, or maternal stress) and their potential to induce epigenetic modifications. Similar to GWAS, epigenome-wide association studies (EWAS) aggregate epigenetic markers across the whole epigenome. A large EWAS that tested numerous cytosine-phosphate-guanine nucleotide base pairings (CpGs) for an association with left-handedness was recently published^17^. Meta-analysis of 3914 whole-blood samples from adult subjects showed that left-handedness was associated with CpGs located nearby SNPs that are known to explain phenotypic variance in handedness. However, overall, very little variance was explained by DNA methylation. Given that the authors also reported temporal instability of associations across different types of tissue, it was concluded that brain tissue rather than peripheral one may be better suited for future approaches.

As already introduced above, unraveling factors that play a role in the ontogenesis of asymmetries may also have clinical relevance. Non-right-handedness (i.e., left-handedness and both-handedness/mixed-handedness) as well as atypical lateralization of other forms of functional asymmetries has been extensively studied in relation to neurodevelopmental, psychiatric, and mental disorders. Amongst others, a heightened prevalence of non-right-handedness was found in schizophrenia^31,32^, dyslexia^33,34^, and autism spectrum disorder (ASD)^35^. Recent meta-analyses further confirmed this pattern for post-traumatic stress disorder (PTSD)^36^, but not for depression^37^. Still, for depression^38^ as well as for schizophrenia^32,39,40^, and dyslexia^41,42^, studies have shown a higher frequency of atypical language lateralization. Since stress is considered a crucial factor in the ontogenesis and progression of such disorders, it has been argued that disturbed asymmetries may mediate the development of psychopathological outcomes in diathesis-stress models^43^. However, it remains debated how far specific alterations in lateralization represent a distinct diagnostic feature of certain mental disorders and how atypical lateralization does relate to observed symptoms. In this context, Mundorf et al.^9^ discuss three different kinds of associations that may characterize the relation between atypical asymmetries and psychopathological outcomes: (a) There are factors that simultaneously contribute to diffuse atypical lateralization on a whole-brain level and to a generic risk for psychopathology in a transdiagnostic way (non-specific association). (b) There are factors that contribute to function-specific atypical lateralization and to a risk for a specific diagnosis (diagnosis-specific association). (c) There are factors that contribute to a specific symptomatology in a transdiagnostic way on the level of lateralization as well as on the level of psychopathology (symptom-specific association). Importantly, these three feasible associations pose different predictions for atypical lateralization patterns as observed across different mental disorders that should be tested by means of empirical research^9^.

One severe limitation that does apply to the majority of cited studies is the issue of shallow phenotyping. As we outlined in more depth in a recent opinion paper^3^, shallow phenotyping can be understood as a waiver to conceptualize (dimension of conceptualization) and to measure (dimension of measurement) a phenotype with sufficient complexity. Regarding the dimension of conceptualization in the case of functional asymmetries, it becomes apparent that most studies only assess handedness but largely neglect other forms of functional lateralization. However, other kinds of functional asymmetries may be better suited than handedness for some research interests. For instance, we endorsed the integration of social laterality phenotypes such as hugging since these allow to capture different evolutionary pressures and may be better suited for research across species^3^. From a dimension of measurement, it may be claimed that handedness, the most common proxy for hemispheric asymmetries, is commonly not assessed accurately. In contrast, handedness is often only deduced from a unidimensional measure. In its most extreme, this rationale can be found in the assessment of handedness in terms of only one item that typically asks for writing hand. Thereby handedness is treated as a binary concept and only refers to one manual task (i.e., writing). Of note, many studies in the field of laterality have recognized this issue and satisfy an assessment of handedness using several items. For instance, many researchers use the Edinburgh Handedness Inventory (EHI^44^), a questionnaire that queries hand preference for various manual tasks (e.g., handling a knife, brushing teeth). Such an approach ultimately allows the calculation of a laterality quotient (LQ) which satisfies handedness as a continuous variable. Still, for the EHI as well as for the simple assessment of writing hand, research broadly relies on self-reported preference measures. Only a few studies further integrate performance measures that assess hand skill. A promising example of such a performance measure is the Pegboard task^45^, which requires participants to place several pegs initially stuck in a straight row of holes on a board in a second parallel row of other holes as quickly as possible. As this is done with both hands consecutively while reaction time is taken, it is possible to compare performance of the left and the right hand. Using the Pegboard task rather than a shallow handedness phenotype has resulted in the identification of the first genetic variants associated with handedness in GWAS^46^. Ideally, to come to a preferably differentiated picture of an individual’s lateralization, studies may combine different measures of self-reported hand preference and measures of hand skill as well as measures of other forms of functional asymmetries (e.g., language lateralization). This notion may be especially relevant considering that different performance measures of handedness (the Pegboard task amongst them) have been revealed to show only small correlations among each other and may reflect distinct dimensions of asymmetries^47^. Undoubtedly, a comprehensive assessment of functional asymmetries—which we refer to as deep phenotyping—may not be applicable for larger-scale studies. However, even though the rigid focus on handedness as a sole proxy for functional asymmetries alongside its unidimensional measurement may have become some kind of common minimal standard, a deeper phenotyping of functional lateralization may be indicated to achieve further progress in laterality research^3^.

Capturing functional lateralization phenotypes by means of deep phenotyping, this study aims to further accumulate knowledge on how environmental factors (i.e., birth factors) play a role in the ontogenesis of handedness and other forms of functional lateralization. Therefore, the first part of the study may be considered a replication approach of the findings by de Kovel et al.^24^. Second, we aimed at understanding associations between handedness and other forms of functional lateralization with subclinical tendencies of several mental disorders in a healthy sample.

## Materials and methods

### Sample

We recruited healthy participants between 18 and 35 years with German language skills sufficient for understanding questionnaires and instructions given in our study. Moreover, all participants were of Central European ancestry. Ancestry was assessed by means of self-report inquiring the country of descent of participants as well as all parents and grandparents. Individuals reporting Central European ancestry for all three generations were eligible to participate. In our definition, Central European ancestry covered all Northern, Western and Southern Europe, including Spain, while Portuguese descent was excluded. We included Polish and Russian ancestry, but excluded individuals of Southeast European descent (i.e., Turkey and Greece). With respect to handedness, we had no specific inclusion and exclusion criteria but we aimed for a balanced ratio of all handedness categories so that we specifically enrolled left- and mixed-handed individuals. Thus, we over-selected participants with atypical handedness in order to improve statistical power and approach variance homogeneity in statistical analyses. Study advertisement only indicated that the study investigated handedness and did not reference handedness and mental health. Since we also excluded participants reporting psychopathological conditions, we do not believe the results of the current study to be biased by participant recruitment and advertising. In total, we tested *N* = 631 participants.

This study was approved by the local ethics committee of the Faculty of Psychology at Ruhr University Bochum, Bochum, Germany. All participants gave written informed consent and were treated in accordance with the declaration of Helsinki.

### Procedure

Data collection took place between 11/04/2018 and 14/10/2022. Having given informed consent, participants completed an online survey asking for above-mentioned inclusion and exclusion criteria as well as for several factors surrounding their birth (e.g., birth weight, mother’s health, breastfeeding). Eligible participants were then invited for testing at Ruhr University Bochum. Testing sessions started with a second online survey including the Edinburgh Handedness Questionnaire (EHI^44^) and the Waterloo Footedness Questionnaire (WFQ^48^) as self-report asymmetry measures of handedness and footedness, respectively. Moreover, participants completed validated German versions of the following clinical questionnaires: the Beck’s Depression Inventory (BDI; English original^49^; German version^50^), the Adult ADHD Self-Report Scale Symptom Checklist (ASRS -v1.1; English original^51^; German version^52^), the State-Trait Anxiety Inventory—Trait (STAI-T; English original^53^; German version^54^), the Childhood Trauma Questionnaire (CTQ; English original^55^; German version^56^) and the Schizotypal Personality Questionnaire (SPQ; English original^57^; German version^58^). Finally, participants performed various hand skill tasks including the Pegboard task^45^, the Alphabet test^59^, and the Tapley–Bryden test^60^.

Moreover, language lateralization was assessed using a Dichotic listening task (DLT^61^) and lateralization for visual attention/visuo-spatial perception was assessed using a Line bisection task^62^. At the end of the testing session, participants were compensated with 20 euros or course credit.

### Data cleaning and data aggregation

Data cleaning is described in detail in the supplementary material (Methods section).

Laterality quotients (LQs) were calculated by means of the following formula: LQ = [(right − left)/(right + left)] × 100. Using the EHI and the WFQ to create categories of left-, mixed-, and right-handedness/-footedness, we defined scores of < = − 60 as left-handed/left-footed and scores of > = + 60 as right-handed/right-footed. Participants scoring between these cutoffs were classified as mixed-handed/mixed-footed. Behavioral asymmetry tasks (e.g., Alphabet test, Line bisection) as well as clinical questionnaires were analyzed according to corresponding manuals.

### Statistical analysis

Statistical analysis was conducted in R version 4.1.2 (2021-11-01) and RStudio. The manuscript was prepared using the papaja package^63^. R scripts used for analysis can be retrieved from the Open Science Framework (https://osf.io/nkem6/).

We grouped measured variables in three conceptual categories: (1) birth factors, (2) asymmetry measures, and (3) clinical questionnaires. Quantitative asymmetry measures, birth factors, and clinical questionnaire scores were transformed to normality using the bestNormalize() function^64^, which tests different normalizing procedures and applies the one with the best outcome. For details, see supplementary material (“Methods” section, Figs. S1 to S6). Due to intercorrelations of the variables (shown in the supplementary material, Figs. S7 to S9), the number of effective tests was estimated using the meff() function from the poolr package^65^ for each set of variables. Table 1 summarizes variables included in our statistical analysis after data transformation including number of effective tests.

**Table 1:** Overview over asymmetry measures, birth factors, and clinical questionnaires included as predictors or outcomes in our statistical analysis alongside their scale level (binary/categorical vs. quantitative).

	Asymmetry measures		Birth factors		Clinical questionnaires
	Quantitative	Categorical	Quantitative	Binary	Quantitative
1	EHI LQ	EHI L–M–R	Birth month	Any substances	BDI
2	WFQ LQ	WFQ L–M–R	Birth year	Any health problems	ASRS
3	Pegboard LQ		Maternal age at birth	Any birth complications	STAI-T
4	Alphabet LQ		Birth order position	Breastfeeding	CTQ
5	Tapley LQ		Birth weight	Twin birth	SPQ
6	DLT LQ			Firstborn	
7	Line bisection				
M	7	2	5	6	5
M_eff_	5	1	4	5	3

*EHI* Edinburgh Handedness Questionnaire, *LQ* Laterality quotient, *WFQ* Waterloo Footedness Questionnaire, *DLT* Dichotic listening task, *L–M–R* left-mixed-right, *BDI* Beck’s Depression Inventory, *ASRS* Adult ADHD Self-Report Scale Symptom Checklist, *STAI-T* State-Trait Anxiety Inventory—Trait, *CTQ* Childhood Trauma Questionnaire, *SPQ* Schizotypal Personality Questionnaire, *M* number of measured variables, *M*_*eff*_ Since measured variables within one set are highly intercorrelated (Figs. S7 to S9), the number of effective tests was estimated using the meff() function from the poolr package.

We applied different statistical models to analyze hypothesized relations between these variables. After descriptive statistics (Part 1), we modeled asymmetry measures as a function of birth factors (Parts 2–5). Subsequently, we modeled clinical questionnaires as a function of asymmetry measures (Parts 6 and 7). Therefore, different variables (binary/categorical vs. quantitative) served either as predictor or outcome variables (Table 2). FDR correction was applied to adjust for multiple comparisons using the product of the number of effective tests for predictors and outcomes (e.g., in Part 2, we applied FDR correction for 5 (quantitative asymmetry measures) × 4 (quantitative birth factors) independent tests). For significant effects of ANOVAs concerning non-binary outcome measures, we continued with Bonferroni-corrected pairwise post-hoc tests. In Part 5b, we specifically aimed at replicating the results by de Kovel et al.^24^. We did so by modeling writing hand measured by the first EHI item (left vs. right, excluding mixed-handers) as a function of significant predictor variables in the study by de Kovel et al.^24^, specifically birth weight, birth size, breastfeeding, twin status, and the presence of any birth complication.

**Table 2:** Overview over the statistical models we applied to account for different effective directions between the measured variables (predictors vs. outcomes) alongside their scale level (binary/categorial vs. quantitative).

Part of analysis	Predictors	Outcomes	Statistical model	*M*_*eff*_
Part 1	Descriptive sample characteristics			
Part 2	Birth factors (quantitative)	Asymmetry measures (quantitative)	Linear regression	20
Part 3	Birth factors (binary)	Asymmetry measures (quantitative)	ANOVA	25
Part 4	Birth factors (quantitative)	Asymmetry measures (categorical)	ANOVA	4
Part 5a	Birth factors (binary)	Asymmetry measures (categorical)	Chi-squared test	5
Part 5b	Birth factors (quantitative and binary)	Writing hand L–R (binary)	Logistic regression	
Part 6	Asymmetry measures (quantitative)	Clinical questionnaires (quantitative)	Linear regression	15
Part 7	Asymmetry measures (categorical)	Clinical questionnaires (quantitative)	ANOVA	3

*M*_*eff*_ effective number of tests used for FDR correction. Equals the product of the effective number of tests determined separately for predictors and outcomes (see Table 1).

We checked statistical assumptions of nominally significant (*p* < 0.05) linear regression models (Parts 2 and 6) by means of visual inspection of the following residual plots: correct specification of the model (residuals vs. fitted values), normality of residuals (normal Q–Q), homoscedasticity of residuals (scale location) and existence of outliers or influential data points (residuals vs. leverage).

For nominally significant (*p* < 0.05) ANOVAs (Parts 3, 4, and 7), we checked required assumptions by means of visual inspection of the following residual plots: correct specification of the model (residuals vs. fitted values) and normality of residuals (normal Q-Q). Moreover, we performed Levene’s tests in order to test for homogeneity of variance.

Models in which at least one of the required assumptions seemed to be violated were excluded so that in the following sections, we only discuss regression models producing significant results while fulfilling all required assumptions.

## Results

### Part 1: descriptive sample characteristics

The final sample comprised *N* = 598 participants (72.58% female) born between 1984 and 2003. Mean age was 23.61 years (*SD* = 3.88, range = 18–35). On average, participants had 13.51 years of education (*SD* = 2.99, range = 4–23).

Table 3 shows the descriptive statistics of the quantitative laterality indices, quantitative birth factors, and clinical questionnaires for the final sample. Descriptive statistics for the categorical asymmetry measures and birth factors can be found in Table 4.

**Table 3:** Descriptives of quantitative variables: laterality indices, quantitative birth factors, and clinical questionnaires.

	*N*	*M*	*SE*	Median	Min	Max	Range
EHI LQ	598	49.43	70.70	88.24	− 100.00	100.00	200.00
WFQ LQ	598	35.85	51.88	46.67	− 100.00	100.00	200.00
Peg LQ	598	1.50	5.49	1.86	− 23.61	18.90	42.51
Alphabet LQ	598	18.85	27.91	31.30	− 51.93	56.80	108.73
Tapley LQ	598	9.60	14.79	14.56	− 32.89	45.00	77.89
DLT LQ	598	9.34	23.49	9.09	− 76.00	100.00	176.00
Line bisection	598	− 18.85	39.54	− 18.40	− 169.59	125.07	294.66
Birth month	598	6.66	3.48	7.00	1.00	12.00	11.00
Birth year	598	1995.55	4.14	1996.00	1984.00	2003.00	19.00
Maternal age at birth	596	30.38	4.44	30.00	17.00	42.00	25.00
Birth weight (g)	557	3353.18	573.15	3390.00	1220.00	5400.00	4180.00
Birth size (cm)	551	51.37	3.24	52.00	33.00	64.00	31.00
BDI	598	5.54	5.23	4.00	0.00	32.00	32.00
ASRS	598	43.33	8.90	43.00	20.00	70.00	50.00
STAI-T	598	37.09	9.73	36.00	20.00	69.00	49.00
CTQ	598	34.04	9.76	31.00	25.00	91.00	66.00
SPQ	598	14.89	10.45	13.00	0.00	53.00	53.00

**Table 4:** Descriptives of categorical variables: laterality indices, quantitative birth factors, and clinical questionnaires.

Variable	*N*	*N* Left	*N* Mixed	*N* Right	*N* No	*N* Yes	Missing
EHI L–M–R	598	101	82	415			0
WFQ L–M–R	598	60	309	229			0
Any substances	598				511	87	0
Any health problems	598				488	110	0
Any complications	598				368	230	0
Breastfeeding	597				96	501	1
Twin birth	598				578	20	0
Firstborn	598				311	287	0

When applying a L–M–R handedness categorization based on the EHI, 101 participants (17%) were left-handed (EHI LQ < − 60), 82 participants (14%) were mixed-handed (− 60 < EHI LQ < 60), and 415 participants (69%) were right-handed (EHI LQ > 60) (Table 4). In a L–R dichotomization, 142 participants (24%) were left-handed (EHI LQ < = 0) and 456 participants (76%) were right-handed (EHI LQ > 0).

Based on the WFQ, 60 (10%), 309 (52%), and 229 (38%) participants were left- (WFQ LQ < − 60), mixed- (− 60 < WFQ LQ < 60), and right-footed (WFQ LQ > 60), respectively (Table 4). In the L–R format, 115 participants (19%) were left- (WFQ LQ < = 0) and 483 participants (81%) were right-footed (WFQ LQ > 0).

Table 5 shows mean values and standard deviations as well as results from *t*-tests for quantitative birth factors and clinical questionnaires between left- (EHI LQ < = 0) and right-handers (EHI LQ > 0). Table 6 shows the same information for left- (EHI LQ < − 60) vs. mixed- (− 60 < EHI LQ < 60) vs. right-handers (EHI LQ > 60).

**Table 5:** Quantitative birth factors and clinical questionnaires (untransformed) in left- (EHI LQ < = 0) vs. right-handers (EHI LQ > 0).

Dependent variable	*t*	df	*p*	Cohen’s *d*	95% CI		Left-handers		Right-handers	
					Lower	Upper	*M*	*SD*	*M*	*SD*
Birth month	0.82	232.45	0.411	0.08	− 0.11	0.27	6.72	3.47	6.44	3.52
Birth year	2.11	236.56	0.036	0.20	0.01	0.39	1995.75	4.14	1994.92	4.11
Maternal age at birth	− 1.41	212.84	0.159	− 0.15	− 0.33	0.04	30.23	4.28	30.87	4.89
Birth weight	0.25	214.00	0.803	0.03	− 0.17	0.22	3356.65	568.11	3342.11	590.99
Birth size	− 0.71	221.82	0.476	− 0.07	− 0.27	0.13	51.32	3.26	51.55	3.18
BDI	− 0.19	273.09	0.849	− 0.02	− 0.21	0.17	5.52	5.42	5.61	4.60
ASRS	− 0.92	278.12	0.357	− 0.08	− 0.27	0.11	43.16	9.24	43.87	7.71
STAI-T	− 0.59	250.40	0.553	− 0.05	− 0.24	0.13	36.96	9.89	37.50	9.21
CTQ	0.82	246.90	0.411	0.08	− 0.11	0.27	34.22	9.88	33.47	9.35
SPQ	− 1.34	242.33	0.183	− 0.13	− 0.31	0.06	14.58	10.53	15.89	10.17

*p* values are uncorrected.

**Table 6:** Quantitative birth factors and clinical questionnaires (untransformed) in left- (EHI LQ < − 60) vs. mixed- (− 60 < EHI LQ < 60) vs. right-handers (EHI LQ > 60).

Dependent variable	*F*	*p*	*η*^2^	Right-handers		Mixed-handers		Left-handers	
				*M*	*SE*	*M*	*SE*	*M*	*SE*
Birth month	1.12	0.328	0.00	6.79	0.17	6.24	0.38	6.43	0.35
Birth year	0.99	0.372	0.00	1995.70	0.20	1995.42	0.46	1995.07	0.41
Maternal age at birth	4.21	0.015	0.01	30.27	0.22	29.62	0.49	31.44	0.44
Birth weight	0.39	0.680	0.00	3343.98	29.28	3404.89	63.75	3346.03	59.82
Birth size	1.00	0.370	0.00	51.24	0.17	51.72	0.36	51.60	0.34
BDI	1.23	0.294	0.00	5.41	0.26	6.38	0.58	5.37	0.52
ASRS	1.38	0.252	0.00	43.07	0.44	44.84	0.98	43.14	0.88
STAI-T	0.58	0.563	0.00	36.88	0.48	38.15	1.08	37.09	0.97
CTQ	3.87	0.021	0.01	34.08	0.48	36.17	1.07	32.16	0.97
SPQ	3.41	0.034	0.01	14.32	0.51	17.60	1.15	15.04	1.04

*p* values are uncorrected.

### Part 2: asymmetry ~ quantitative birth factors

**Figure 1:**
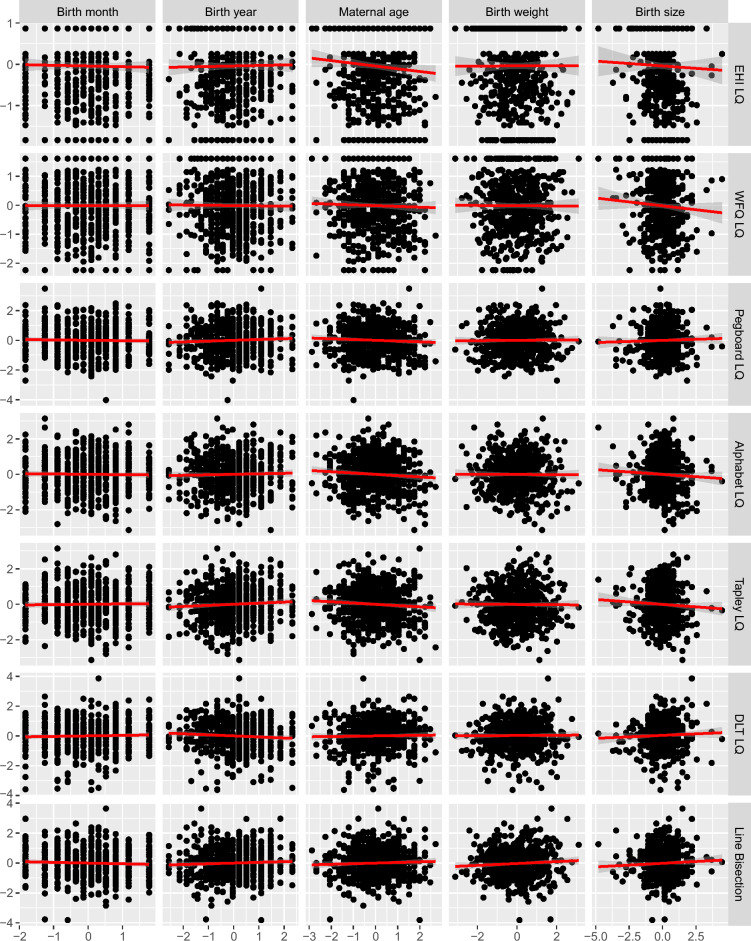
Quantitative asymmetry measures as a function of quantitative birth factors (linear regression).

We ran linear regression models for seven outcomes (quantitative asymmetry measures) and five predictors (quantitative birth factors), applying FDR correction for 20 independent tests (Table 2). Figure 1 and Table S1 show the regression results. None of the models showed a significant association (all *p* > 0.05).

### Part 3: asymmetry ~ binary birth factors

**Figure 2:**
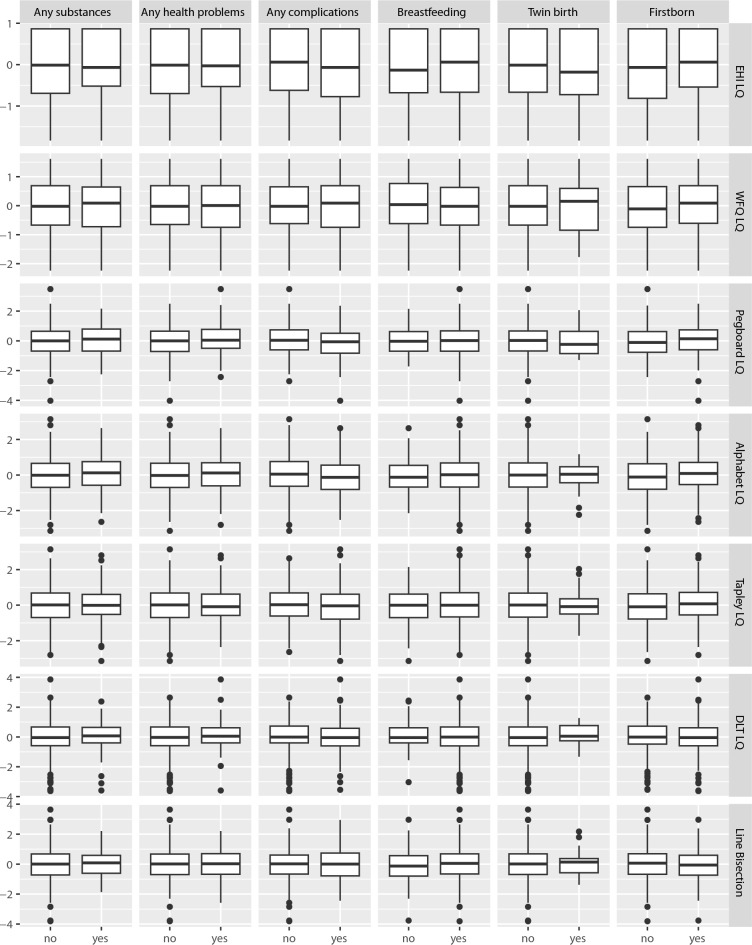
Quantitative asymmetry measures as a function of binary birth factors (ANOVA).

We ran ANOVAs for seven outcomes (quantitative asymmetry measures) and six predictors (binary birth factors), applying FDR correction for 25 independent tests (Table 2). Figure 2 and Table S2 show the ANOVA results. Two models showed a nominally significant association (*p* < 0.05). Pegboard LQ showed weak associations with the variables “any birth complications”, $F(1,596)=4.69$, $p=0.031$, ${\widehat{\eta }}_{G}^{2}=0.008$, 90% CI $[0.000,\, 0.024]$ and “firstborn”, $F(1,596)=4.73$, $p=0.030$, ${\widehat{\eta }}_{G}^{2}=0.008$, 90% CI $[0.000,\, 0.024]$. In particular, participants who reported to have experienced birth complications showed lower Pegboard LQs (i.e., more leftward lateralization) than participants who reported to have experienced no birth complications (Table S2, model 17). On the contrary, participants who reported being firstborn scored higher on the Pegboard LQ (i.e., more rightward lateralization) than participants who reported another birth order position (Table S2, model 38). However, none of these models remained significant after FDR correction.

### Part 4: asymmetry ~ quantitative birth factors

**Figure 3:**
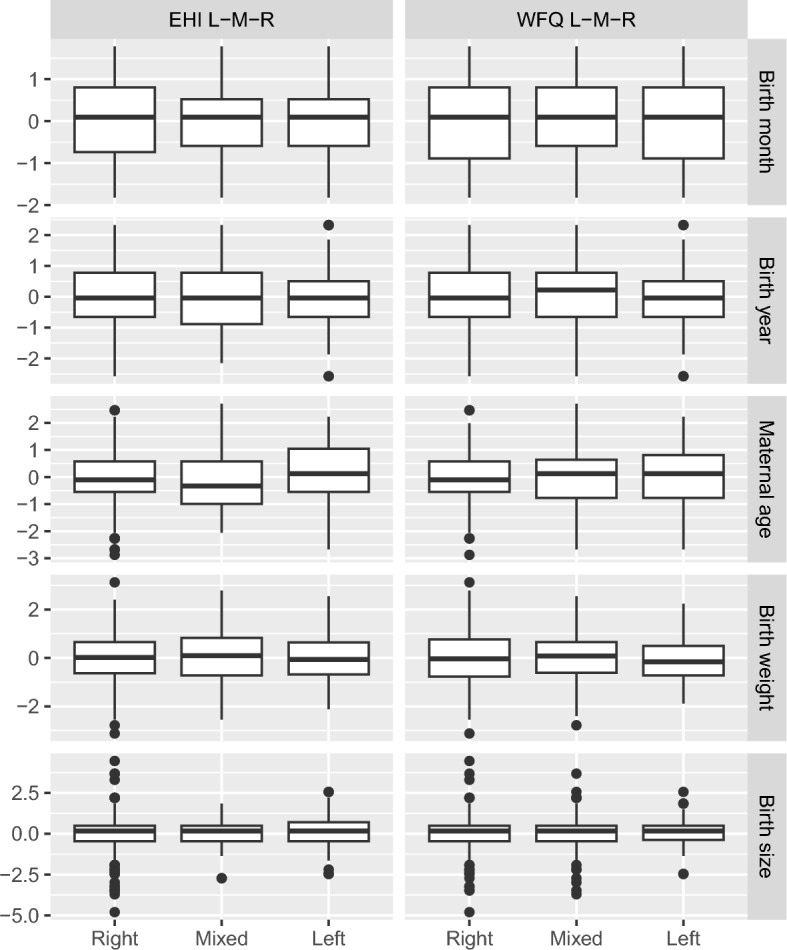
Categorical asymmetry measures (L–M–R) as a function of quantitative birth factors.

We ran ANOVAs for two outcomes (categorical asymmetry measures) and seven predictors (quantitative birth factors), applying FDR correction for 4 independent tests (Table 2). Figure 3 and Table S3 show the ANOVA results. Maternal age at birth showed a nominally significant association with the EHI categories, $F(2,593)=4.28$, $p=0.014$, ${\widehat{\eta }}_{G}^{2}=0.014$, 90% CI $[0.002,\, 0.032]$. Left-handed participants reported higher maternal age at birth compared to mixed- (*p* = 0.017) and right-handed participants (*p* = 0.049). Right- and mixed-handed participants did not differ from each other regarding maternal age at birth (Table S3, model 5). This model did not remain significant after FDR correction (*p* = 0.056).

### Part 5: asymmetry ~ binary birth factors

For Part 5a, we ran Chi square tests for two outcomes (categorical asymmetry measures) and six predictors (binary birth factors), applying FDR correction for 5 independent tests (Table 2). Full results are shown in Table S4. The EHI LQ categories showed a nominally significant association with the variable “firstborn”, Chi square (2, *N* = 598) = 6.21, *p* = 0.045, *V* = 1.76, but did not survive FDR correction (*p* = 0.224).

Moreover, in Part 5b, we aimed to replicate the results by de Kovel et al.^24^ by running a logistic regression analysis on a binary handedness measure (i.e., writing hand, the first EHI item). This analysis included *N* = 150 left-handers and *N* = 435 right-handers (*N* = 13 mixed-handers were excluded from this analysis). Birth weight, birth size, breastfeeding, twinning, and the presence of any birth complications were included as predictors. None of the predictors reached significance (Table 7).

**Table 7:** Replication of de Kovel et al.^24^.

Predictor	*b*	95% CI	*z*	*p*
Intercept	− 1.12	[− 1.68, − 0.60]	− 4.10	< 0.001
Birth weight	− 0.04	[− 0.32, 0.23]	− 0.31	0.757
Birth size	0.15	[− 0.12, 0.42]	1.08	0.281
Breastfeeding	0.01	[− 0.52, 0.57]	0.04	0.967
Twinning	− 0.04	[− 1.23, 1.01]	− 0.07	0.948
Any birth complications	0.19	[− 0.23, 0.60]	0.88	0.381

### Part 6: clinical questionnaires ~ asymmetry

**Figure 4:**
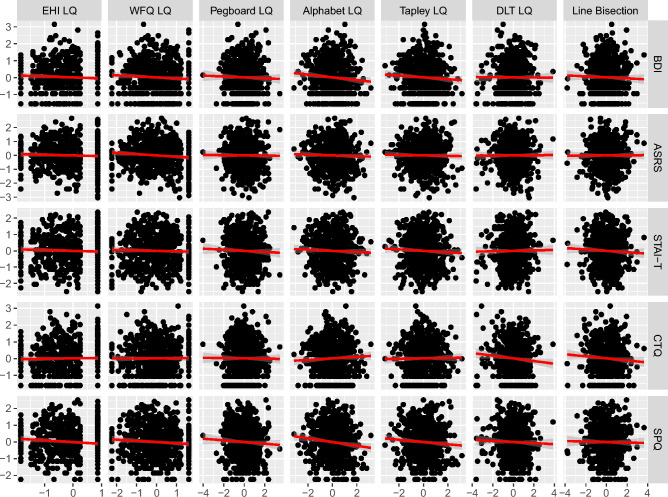
Clinical questionnaires (overall scores) as a function of quantitative asymmetry measures (linear regression).

We ran linear regression models for five outcomes (clinical questionnaires) and seven predictors (quantitative asymmetry measures), applying FDR correction for 15 independent tests (Table 2). Figure 4 and Table S5 show the regression results. The strongest association was found between the Alphabet LQ and the SPQ score, $b=-\,0.11$, 95% CI $[-\,0.19,\,-\,0.03]$, $t(596)=-\,2.73$, $p=0.006$. A nominally significant association was also found between the EHI LQ and the SPQ score, $b=-\,0.10$, 95% CI $[-\,0.20,\,-\,0.01]$, $t(596)=-\,2.22$, $p=0.027$. For both asymmetry measures, higher SPQ scores were associated with lower Alphabet LQ and EHI LQ scores (i.e., more leftward lateralization, Table S5, models 20 and 5). Both did not remain significant after FDR correction (*p* = 0.090 and *p* = 0.129, respectively).

Moreover, there were weak nominally significant associations between BDI score and Alphabet LQ, $b=-\,0.08$, 95% CI $[-\,0.16,\,0.00]$, $t(596)=-\,2.04$, $p=0.042$, CTQ score and DLT LQ, $b=-\,0.08$, 95% CI $[-\,0.16,\,0.00]$, $t(596)=-\,2.03$, $p=0.043$, and between ASRS score and WFQ LQ, $b=-\,0.09$, 95% CI $[-\,0.17,\,-\,0.01]$, $t(596)=-\,2.16$, $p=0.031$. Again, higher scores on the clinical questionnaires were associated with lower LQs for the different asymmetry measures (i.e., more leftward lateralization, Table S5, models 16, 29, and 7, all FDR-corrected *p* = 0.129).

### Part 7: clinical questionnaires ~ asymmetry

**Figure 5:**
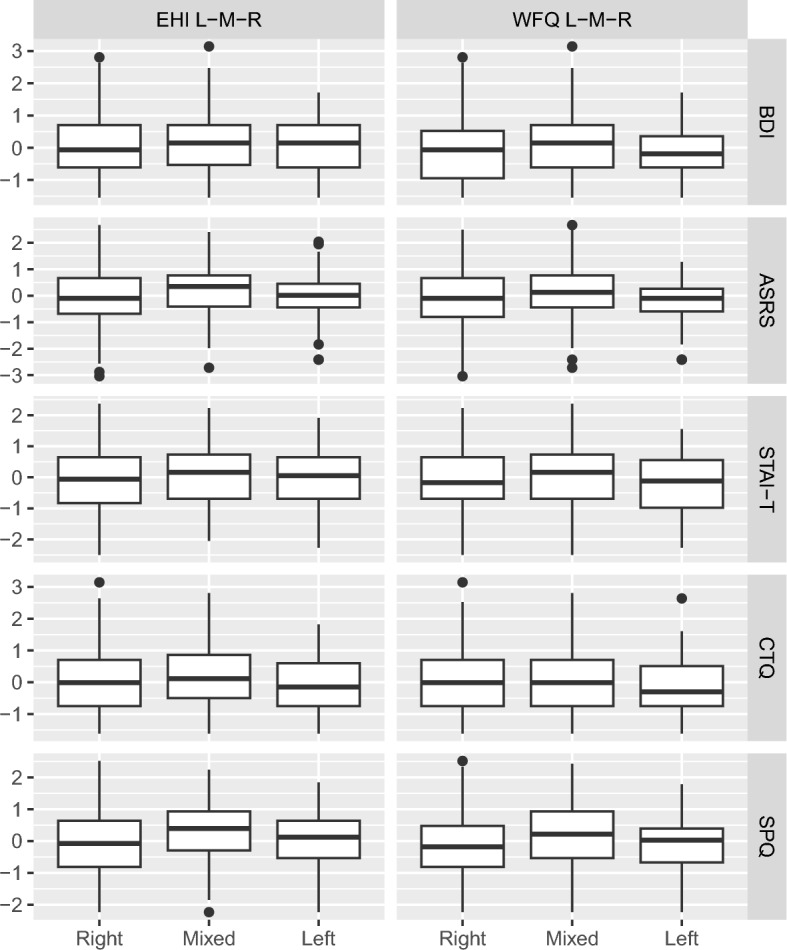
Clinical questionnaires (overall scores) as a function of categorical asymmetry measures (L–M–R) (linear regression).

Finally, we ran ANOVAs for five outcomes (clinical questionnaires) and two predictors (categorical asymmetry measures), applying FDR correction for 3 independent tests (Table 2). Figure 5 and Table S6 show the ANOVA results. The WFQ categories showed an association with the ASRS score, $F(2,595)=7.28$, $p=0.001$, ${\widehat{\eta }}_{G}^{2}=0.024$, 90% CI $[0.007,\,0.046]$. However, the significant Levene’s test (*p* = 0.015) indicated violation of the assumption of variance homogeneity (Table S6, model 7, FDR-corrected *p* = 0.003).

Moreover, the WFQ categories were associated with the SPQ score, $F(2,595)=4.70$, $p=0.009$, ${\widehat{\eta }}_{G}^{2}=0.016$, 90% CI $[0.002,\,0.034]$, in that mixed-footed participants scored significantly higher than right-footed participants (*p* = 0.011). The WFQ categories further showed associations with the STAI-T score, $F(2,595)=3.46$, $p=0.032$, ${\widehat{\eta }}_{G}^{2}=0.012$, 90% CI $[0.001,\,0.028]$ and the BDI score, $F(2,595)=3.07$, $p=0.047$, ${\widehat{\eta }}_{G}^{2}=0.010$, 90% CI $[0.000,\,0.026]$, but none of the post-hoc tests reached significance (Table S6, models 10, 8, and 6). All associations remained significant after FDR correction (FDR-corrected *p* = 0.013, 0.024, and 0.028, respectively).

The EHI categories showed an association with the SPQ score, $F(2,595)=3.82$, $p=0.022$, ${\widehat{\eta }}_{G}^{2}=0.013$, 90% CI $[0.001,\,0.030]$, with mixed-handed participants reporting significantly higher SPQ scores than right-handed participants (*p* = 0.021) (Table S6, model 5, FDR-corrected *p* = 0.022).

To sum up, effects for ANOVAs on clinical questionnaires as a function of categorial asymmetry measures seem to be driven by the middle (mixed-handed/mixed-footed) category mainly.

## Discussion

In this study, we considered associations between functional hemispheric asymmetries and early life factors as well as subclinical symptoms of psychopathology in healthy participants. This triad is interesting from a conceptual point of view: it relates to the idea that the ontogenesis of functional hemispheric asymmetries may be disturbed by certain birth factors resulting in atypical lateralization that sets a greater vulnerability or risk for psychopathological outcomes, or actually mediates their development^43^.

Previous studies such as de Kovel et al.^24^ relied on handedness as a single, categorical index of functional asymmetries which was inquired with only one item asking for hand preference. In this regard, the current study is unique since different forms of functional hemispheric asymmetries (i.e., handedness, footedness, language lateralization, visuo-spatial perception) were approached by means of deep phenotyping with different measures (i.e., self-report questionnaires, tests measuring dexterity, DLT and Line bisection task). Birth factors and clinical questionnaires were assessed by means of self-report. Thereby, our approach allowed for more nuanced statistical tests and also acknowledged the fact that different laterality phenotypes may differ in their strength of association with our predictors (e.g., early life factors and mental health outcomes) as well as in their sensitivity for showing these associations in our statistical analyses.

Few associations reached statistical significance and most did not survive correction for multiple testing. In Parts 2 to 5, we modelled birth factors as a function of functional asymmetries but did not find any significant effects in Part 2. For Part 3, we found the occurrence of birth complications and higher birth order position to model the LQ as calculated for the Pegboard task in that birth complications and higher birth order position were associated with lower Pegboard LQs (i.e., more leftward lateralization). For Part 4, handedness categories (left-handed/mixed-handed/right-handed) were identified as a function of maternal age at birth in that left-handed participants reported higher maternal age at birth as the other two handedness categories (which did not differ amongst each other). For Part 5a, prevalence of handedness categories (left-handed/mixed-handed/right-handed) significantly differed between participants of different birth order positions. In Parts 6 and 7, we modelled functional asymmetries as a function of clinical questionnaires. For Part 6, scores on clinical questionnaires were identified as a function of LQs as calculated for different tasks. The EHI LQ as well as the Alphabet LQ were shown to model the SPQ score in that higher SPQ scores were associated with lower LQs in these tasks (i.e., more leftward lateralization). Similarly, higher BDI scores predicted lower Alphabet LQs while higher CTQ scores predicted lower DLT LQs, and higher ASRS scores predicted lower WFQ LQs. Therefore, in Part 6, higher scores on clinical questionnaires were uniformly predicted by more leftward lateralization. For Part 7, we found few significant differences for categorical asymmetry measures with respect to clinical questionnaires, which remained significant after correction for multiple testing. In detail, mixed-footed (WFQ) as well as mixed-handed (EHI) participants showed higher SPQ scores than right-footed (WFQ) and right-handed (EHI) participants. Hence, these effects seemed to be driven by the middle (mixed-footed/mixed-handed) category in large parts. The WFQ was further associated with the STAI-T and the BDI scores but post-hoc tests did not reach significance.

Noteworthy, most effects only reached statistical significance when not controlling for multiple comparisons. We consider it important to highlight the small effect sizes of basically all observed effects. That is, eta squared was ${\eta }^{2}$ = 0.01 for most significant associations and ${\eta }^{2}$ = 0.02 at the maximum for differences in the ASRS score and the SPQ score between participants of different handedness categories. Consequently, the associations found to be significant do not explain a considerable part of variance in handedness or other forms of functional lateralization. Along these lines, the great majority of associations tested in the current study did not reach statistical significance at all. Therefore, we did not interpret the significant effects functionally.

The prevailing pattern of a large number of non-effects alongside small effect sizes of nominally significant results in the current study is in accordance with existing literature. Most prominently, de Kovel et al.^24^ also report only few statistically significant associations with negligible effect sizes between adult left-handedness and a plentitude of birth factors. Amongst the few factors that turned out to be significant in the publication by de Kovel et al.^24^, we also assessed birth weight, birth size, breastfeeding, twin status, and birth complications. Therefore, in Part 5b we attempted to directly replicate the findings of de Kovel et al.^24^ in modelling handedness as classified by the writing hand item of the EHI as a function of these predictors. Noteworthy, none of the predictors reached significance. Failure to replicate the results by de Kovel et al.^24^ may be attributed to the fact that our sample size was substantially smaller. Importantly, de Kovel et al.^24^ used large-scale data from the UK Biobank covering ~ 500,000 participants. However, having said that most of the associations tested in our data did not reach statistical significance nor convincing effect sizes, one may conclude that they lack decisive importance at the population level. However, in the larger study by de Kovel et al.^24^ effects reached statistical significance and probably did not so by chance. It is rather conceivable that effects are significant and thus important for the single individual. For instance, being part of a multiple birth may be of great importance in triggering the development of atypical brain asymmetry in some individuals, but not in others.

However, de Kovel et al.^24^ conclude that the current literature does not support the notion that specific environmental variables (in their as well as in our study) may fill the gap between variance explained by genetic factors and so-far unexplained variance in handedness (and other functional asymmetries). This is the case for many clinical phenotypes in the epidemiological literature, for which twin studies show a substantial amount of variance explained by non-shared environmental factors. However, the role of non-shared environmental factors is likely to be heavily overestimated (and overinterpreted), as it is based on simple subtraction; it equals the variance not explained by additive genetic and shared environmental factors. Therefore, what is typically called non-shared environmental variance not only includes measurement error and gene-environment interaction, but also chance or random events^27^. De Kovel et al.^24^ also accounted for this perspective in discussing their findings in the context of randomness in fetal brain development as already elucidated in the introduction. Notably, for the birth factors it has been proposed that their effect is mediated via epigenetic mechanisms. However, a large EWAS found only little handedness variance to be captured by epigenetic modifications of DNA^17^, casting doubt not only on strong associations between birth factors and functional asymmetries, but also on strong associations between epigenetic factors and functional asymmetries.

While de Kovel et al.^24^ only tested for associations with self-reported writing hand, we included diverse functional laterality phenotypes and assessed them by means of deep phenotyping. Since we replicated the gross pattern of non-effects found by de Kovel et al.^24^ for self-reported hand preference, deep phenotyping of multiple asymmetry measures does not seem to enhance the power of unraveling relations for the research question at hand. However, it should be noted that birth factors as well as clinical questionnaires in the current study were also assessed by means of self-report. Self-reports are typically prone to certain biases as well as reporting errors. Especially for the birth factors collected as self-report (i.e., birth weight), we had to exclude several data points based on plausibility (for details, see the “Method” section of the supplementary material). As a consequence, we cannot rule out that a more precise/objective measurement of the included birth factors as well as of clinical symptoms (e.g., by means of clinical interviews) would have led to a different pattern of results.

Moreover, for the current study, it is worth mentioning, that statistical power might have been impeded by the fact that our data consistently violated required assumptions of the appropriate statistical models. To counteract, we applied diverse transformations which did not always lead to perfect distributions. In this regard it seems debatable in how far data on lateralization phenotypes may represent a special case (bearing in mind their often J-shaped distribution).

Similarly, one might question whether the clinical questionnaires used in the current study were actually suitable in our healthy sample. Since participants were only included reporting no mental, psychiatric, or neurological disorder, at best we might have covered preclinical manifestations of the psychopathological constructs. As a result, the variance in our clinical questionnaires might not have been sufficient enough to unravel putatively existing effects. This assumption gains further plausibility considering the fact that clinical samples frequently produce large effects with respect to laterality measures. For instance, as already mentioned in the introduction, meta-analyses univocally confirm a certain relation between atypical handedness or other forms of functional lateralization and diverse clinical diagnoses (e.g., schizophrenia^31,32^, ASD^35^, PTSD^36^). Therefore, one might conclude there is some sort of rubicon covering noticeable qualitative differences between healthy and clinical samples regarding asymmetry measures. Regarding the different kinds of associations that may possibly link atypical functional lateralization and psychopathological outcomes^9^, non-occurrence of effects in the context of our healthy sample may rather point towards a broader, generic and transdiagnostic effect of atypical lateralization on psychopathology, if any.

Still, for the results that reached statistical significance for a categorical operationalization of asymmetry measures in the current study, it is striking that they often concerned the “middle” category. Indeed, effects often seemed to be driven by mixed-handed/mixed-footed participants while more extreme forms of lateralization towards the left or the right side of the continuum did not seem to be influential. Hence, one might speculate that it is not left- but mixed-handedness/-footedness that shows the closest association with clinical constructs. Indeed, this is in line with several meta-analyses suggesting that disorders such as PTSD^36^ and schizophrenia^32^ are related to mixed-handedness in particular, rather than left-handedness. Therefore, it has been put forward that a reduction or an absence of asymmetries (such as mixed-handedness) rather than a reversal (such as left-handedness) is of relevance for clinical outcomes^36^.

In conclusion, the current study further confirms previous findings of mostly negligible associations between birth factors and functional asymmetry measures in healthy individuals. Deep phenotyping did not lead to any substantial changes in this overall results pattern, confirming the robustness of previous findings using shallower phenotyping. Likewise, effects between functional lateralization and diverse psychopathological outcomes did not achieve noticeable predictive power in our sample of healthy individuals. Further research might identify qualitative differences between healthy and clinical samples as studying the latter typically renders strong effects for lateralization indices. Future studies might also benefit from the inclusion of social laterality phenotypes and biological markers as well as from the application of longitudinal approaches.

### Supplementary Information

Supplementary Information.

## Data Availability

Raw data of the current study cannot be provided since this option was not included in the corresponding ethical approval. R scripts used for analysis can be retrieved from from the Open Science Framework (https://osf.io/nkem6/).

## References

[1] Güntürkün O, Ströckens F, Ocklenburg S (2020). Brain lateralization: A comparative perspective. Physiol. Rev..

[2] Versace E, Vallortigara G (2015). Forelimb preferences in human beings and other species: Multiple models for testing hypotheses on lateralization. Front. Psychol..

[3] Pfeifer LS (2022). Broadening the scope: Increasing phenotype diversity in laterality research. Front. Behav. Neurosci..

[4] Frasnelli E (2014). The bee as a model to investigate brain and behavioural asymmetries. Insects.

[5] Ocklenburg S, Hirnstein M, Beste C, Güntürkün O (2014). Lateralization and cognitive systems. Front. Psychol..

[6] Vallortigara G, Rogers LJ (2020). A function for the bicameral mind. Cortex.

[7] Papadatou-Pastou M (2020). Human handedness: A meta-analysis. Psychol. Bull..

[8] Ocklenburg S, Berretz G, Packheiser J, Friedrich P (2021). Laterality 2020: Entering the next decade. Laterality.

[9] Mundorf A, Peterburs J, Ocklenburg S (2021). Asymmetry in the central nervous system: A clinical neuroscience perspective. Front. Syst. Neurosci..

[10] Annett M (1998). Handedness and cerebral dominance: The right shift theory. J. Neuropsychiatry Clin. Neurosci..

[11] McManus IC (1985). Handedness, language dominance and aphasia: A genetic model. Psychol. Med..

[12] Armour JA, Davison A, McManus IC (2014). Genome-wide association study of handedness excludes simple genetic models. Heredity.

[13] Ocklenburg S, Beste C, Güntürkün O (2013). Handedness: A neurogenetic shift of perspective. Neurosci. Biobehav. Rev..

[14] Medland SE (2009). Genetic influences on handedness: Data from 25,732 Australian and Dutch twin families. Neuropsychologia.

[15] Medland SE, Duffy DL, Wright MJ, Geffen GM, Martin NG (2006). Handedness in twins: Joint analysis of data from 35 samples. Twin Res. Hum. Genet..

[16] Cuellar-Partida G (2021). Genome-wide association study identifies 48 common genetic variants associated with handedness. Nat. Hum. Behav..

[17] Odintsova VV (2022). DNA methylation in peripheral tissues and left-handedness. Sci. Rep..

[18] Lasser M, Tiber J, Lowery LA (2018). The role of the microtubule cytoskeleton in neurodevelopmental disorders. Front. Cell. Neurosci..

[19] Penazzi L, Bakota L, Brandt R (2016). Microtubule dynamics in neuronal development, plasticity, and neurodegeneration. Int. Rev. Cell Mol. Biol..

[20] Parma V, Brasselet R, Zoia S, Bulgheroni M, Castiello U (2017). The origin of human handedness and its role in pre-birth motor control. Sci. Rep..

[21] Hepper PG, Shahidullah S, White R (1991). Handedness in the human fetus. Neuropsychologia.

[22] Graham JH (2021). Nature, nurture, and noise: Developmental instability, fluctuating asymmetry, and the causes of phenotypic variation. Symmetry.

[23] McManus IC (2021). Is any but a tiny fraction of handedness variance likely to be due to the external environment?. Laterality.

[24] de Kovel CGF, Carrión-Castillo A, Francks C (2019). A large-scale population study of early life factors influencing left-handedness. Sci. Rep..

[25] Laland KN, Kumm J, van Horn JD, Feldman MW (1995). A gene-culture model of human handedness. Behav. Genet..

[26] Laland KN (2008). Exploring gene-culture interactions: Insights from handedness, sexual selection and niche-construction case studies. Philos. Trans. R. Soc. Lond. Ser. B Biol. Sci..

[27] Davey Smith G (2011). Epidemiology, epigenetics and the ’gloomy prospect’: Embracing randomness in population health research and practice. Int. J. Epidemiol..

[28] de Kovel CGF, Lisgo SN, Fisher SE, Francks C (2018). Subtle left-right asymmetry of gene expression profiles in embryonic and foetal human brains. Sci. Rep..

[29] Ocklenburg S (2017). Epigenetic regulation of lateralized fetal spinal gene expression underlies hemispheric asymmetries. Elife.

[30] Schmitz J, Metz GAS, Güntürkün O, Ocklenburg S (2017). Beyond the genome-towards an epigenetic understanding of handedness ontogenesis. Prog. Neurobiol..

[31] Dragovic M, Hammond G (2005). Handedness in schizophrenia: A quantitative review of evidence. Acta Psychiatr. Scand..

[32] Sommer I, Aleman A, Ramsey N, Bouma A, Kahn R (2001). Handedness, language lateralisation and anatomical asymmetry in schizophrenia. Br. J. Psychiatry.

[33] Eglinton E, Annett M (1994). Handedness and dyslexia: A meta-analysis. Percept. Mot. Skills.

[34] Abbondanza F (2023). Language and reading impairments are associated with increased prevalence of non-right-handedness. Child Dev..

[35] Markou P, Ahtam B, Papadatou-Pastou M (2017). Elevated levels of atypical handedness in autism: Meta-analyses. Neuropsychol. Rev..

[36] Borawski J, Papadatou-Pastou M, Packheiser J, Ocklenburg S (2023). Handedness in post-traumatic stress disorder: A meta-analysis. Neurosci. Biobehav. Rev..

[37] Packheiser J (2021). Handedness and depression: A meta-analysis across 87 studies. J. Affect. Disord..

[38] Gadea M, Espert R, Salvador A, Martí-Bonmatí L (2011). The sad, the angry, and the asymmetrical brain: Dichotic listening studies of negative affect and depression. Brain Cogn..

[39] Bleich-Cohen M, Hendler T, Kotler M, Strous RD (2009). Reduced language lateralization in first-episode schizophrenia: An fMRI index of functional asymmetry. Psychiatry Res..

[40] Weiss EM (2006). Language lateralization in unmedicated patients during an acute episode of schizophrenia: A functional MRI study. Psychiatry Res. Neuroimaging.

[41] Altarelli I (2014). Planum temporale asymmetry in developmental dyslexia: Revisiting an old question. Hum. Brain Mapp..

[42] Norton ES, Beach SD, Gabrieli JDE (2015). Neurobiology of dyslexia. Curr. Opin. Neurobiol..

[43] Berretz G, Wolf OT, Güntürkün O, Ocklenburg S (2020). Atypical lateralization in neurodevelopmental and psychiatric disorders: What is the role of stress?. Cortex.

[44] Oldfield RC (1971). The assessment and analysis of handedness: The Edinburgh Inventory. Neuropsychologia.

[45] Annett M (1970). The growth of manual preference and speed. Br. J. Psychol..

[46] Scerri TS (2011). PCSK6 is associated with handedness in individuals with dyslexia. Hum. Mol. Genet..

[47] Buenaventura Castillo CE, Lynch AG, Paracchini S (2019). Different laterality indexes are poorly correlated with one another but consistently show the tendency of males and females to be more left- and right-lateralised, respectively. R. Soc. Open Sci..

[48] Elias LJ, Bryden MP, Bulman-Fleming MB (1998). Footedness is a better predictor than is handedness of emotional lateralization. Neuropsychologia.

[49] Beck AT, Steer RA, Brown GK (1996). Beck Depression Inventory—Second Edition: Manual.

[50] Hautzinger M, Keller F, Kuhner C (2006). Beck Depressions-Inventar, Revision (BDI-II).

[51] Kessler RC (2005). The world health organization adult ADHD self-report scale (ASRS): A short screening scale for use in the general population. Psychol. Med..

[52] Buchli-Kammermann, J., Corbisiero, S. & Stieglitz, R. D. Screening der aufmerksamkeitsdefizit-/hyperaktivitätsstörung (ADHS) im erwachsenenalter: Validierung der deutschen version der ASRS-v1.1. *Klinische Diagnostik und Evaluation* 219–235 (2011).

[53] Spielberger CD, Gorsuch RL, Lushene RE (1970). Manual for the State-Trait Anxiety Inventory.

[54] Laux L, Glanzmann P, Schaffner P, Spielberger CD (1981). State-trait-angstinventar (STAI): Theoretische grundlagen und handanweisungen.

[55] Bernstein DP (2003). Development and validation of a brief screening version of the childhood trauma questionnaire. Child Abuse Negl..

[56] Klinitzke G, Romppel M, Häuser W, Brähler E, Glaesmer H (2012). Die deutsche version des childhood trauma questionnaire (CTQ)—psychometrische eigenschaften in einer bevölkerungsrepräsentativen stichprobe [the german version of the childhood trauma questionnaire (CTQ): Psychometric characteristics in a representative sample of the general population]. Psychother. Psychosom. Med. Psychol..

[57] Raine A (1991). The SPQ: A scale for the assessment of schizotypal personality based on DSM-III-r criteria. Schizophr. Bull..

[58] Klein C, Andresen B, Jahn T (1997). Erfassung der schizotypen persönlichkeit nach DSM-III-r: Psychometrische eigenschaften einer autorisierten deutschsprachigen Übersetzung des schizotypal personality questionnaire (SPQ) von raine [psychometric assessment of the schizotypal personality according to DSM-III-r criteria: Psychometric properties of an authorized German translation of Raine’s schizotypal personality questionnaire (SPQ)]. Diagnostica.

[59] Provins KA, Magliaro J (1993). The measurement of handedness by preference and performance tests. Brain Cogn..

[60] Tapley SM, Bryden MP (1985). A group test for the assessment of performance between the hands. Neuropsychologia.

[61] Kimura D (1961). Cerebral dominance and the perception of verbal stimuli. Can. J. Psychol..

[62] Hausmann M, Ergun G, Yazgan Y, Güntürkün O (2002). Sex differences in line bisection as a function of hand. Neuropsychologia.

[63] Aust, F. & Barth, M. *papaja: Prepare reproducible APA journal articles with R Markdown* (2022).

[64] Peterson RA (2021). Finding optimal normalizing transformations via bestNormalize. R J..

[65] Cinar O, Viechtbauer W (2022). The poolr package for combining independent and dependent p values. J. Stat. Softw..

