# Supplementary material for: A deep phenotyping approach to assess the association of handedness, early life factors and mental health
Source: Sci Rep. 2023 Sep 15;13:15348. doi: 10.1038/s41598-023-42563-7 (PMC10504248; doi:10.1038/s41598-023-42563-7)
Supplement: Supplementary file 1 — Supplementary Information. [file 41598_2023_42563_MOESM1_ESM.pdf]

A deep phenotyping approach to assess the association of handedness, early life factors and mental health

Lena Sophie Pfeifer[1], Judith Schmitz[2], Maike Schwalvenberg[3], Onur Güntürkün[3], and & Sebastian Ocklenburg[3,4,5]

[1] Cognitive Psychology; Institute of Cognitive Neuroscience; Faculty of Psychology; Ruhr University Bochum; Bochum; Germany

[2] Biological Personality Psychology; Georg-August-University Goettingen; Goettingen; Germany

[3] Biopsychology; Institute of Cognitive Neuroscience; Faculty of Psychology; Ruhr University Bochum; Bochum; Germany

[4] Department of Psychology; Medical School Hamburg; Hamburg; Germany

[5] Institute for Cognitive and Affective Neuroscience; Medical School Hamburg; Hamburg; Germany

Author Note

Lena Pfeifer and Judith Schmitz contributed equally to this work.

Correspondence concerning this article should be addressed to Lena Sophie Pfeifer, Cognitive Psychology; Institute of Cognitive Neuroscience; Faculty of Psychology; Ruhr University Bochum; Bochum; Germany. E-mail: lena.pfeifer@rub.de

A deep phenotyping approach to assess the association of handedness, early life factors and mental health

Supplementary Methods

Data cleaning

We removed individuals reporting impaired visual function without correction (N = 14) or impaired hearing ability without correction ($N = 1$). Concerning German language skills, we excluded individuals who reported having no German academic degree *and* not having started learning German before the age of 18 ($N = 5$). Moreover, we removed individuals reporting a psychiatric disorder ($N = 1$) or a neurological disorder ($N = 12$). We did not exclude individuals reporting fear of heights.

Moreover, we aggregated several variables as follows. Since participants originally indicated all educational degrees they had achieved, we created one variable for the highest degree. Similarly, since participants originally indicated years they spent for each educational degree, we added these years to create one variable representing total educational years. Therefore, we only included educational years for degrees that had been completed.

Since data were collected by means of self-report, we included several plausibility checks. Concerning educational variables, we opted to not consider the following cases (i.e., set to "NA"): (1) cases in which no educational degree at all was stated ($N = 2$), (2) cases in which the number of educational years exceeded the participant's age ($N = 2$), (3) cases in which a university degree was stated without stating any school degree ($N = 5$), (4) cases in which participants indicated having achieved a degree without specifying the corresponding number of years ($N = 5$). Last but not least, in cases where participants stated having reached a high school degree, but did not indicate a primary school degree, we added four years to the number of educational years.

To check plausibility of stated birth years, we summed up the variables indicating birth year and age. While the sum was equal to the year of testing in most cases, there was one clear outlier (1968 + 31 = 1999). As it seemed very likely that numbers were switched (1986 + 31 = 2017), we changed the birth year of the corresponding participant to 1986.

Concerning maternal age at birth, we excluded one case where mother's age was indicated by 59. Similarly, we did not include data on birth size exceeding 70 cm.

Transformations of asymmetry measures

Normality of quantitative asymmetry measures, birth factors, and clinical questionnaire scores was tested using the Shapiro-Wilk test for normality. Measures were transformed to normality using the bestNormalize() function (Peterson, 2021), which tests different normalizing procedures and applies the one with the best outcome.

Figure S1 shows the seven quantitative asymmetry measures before transformation:

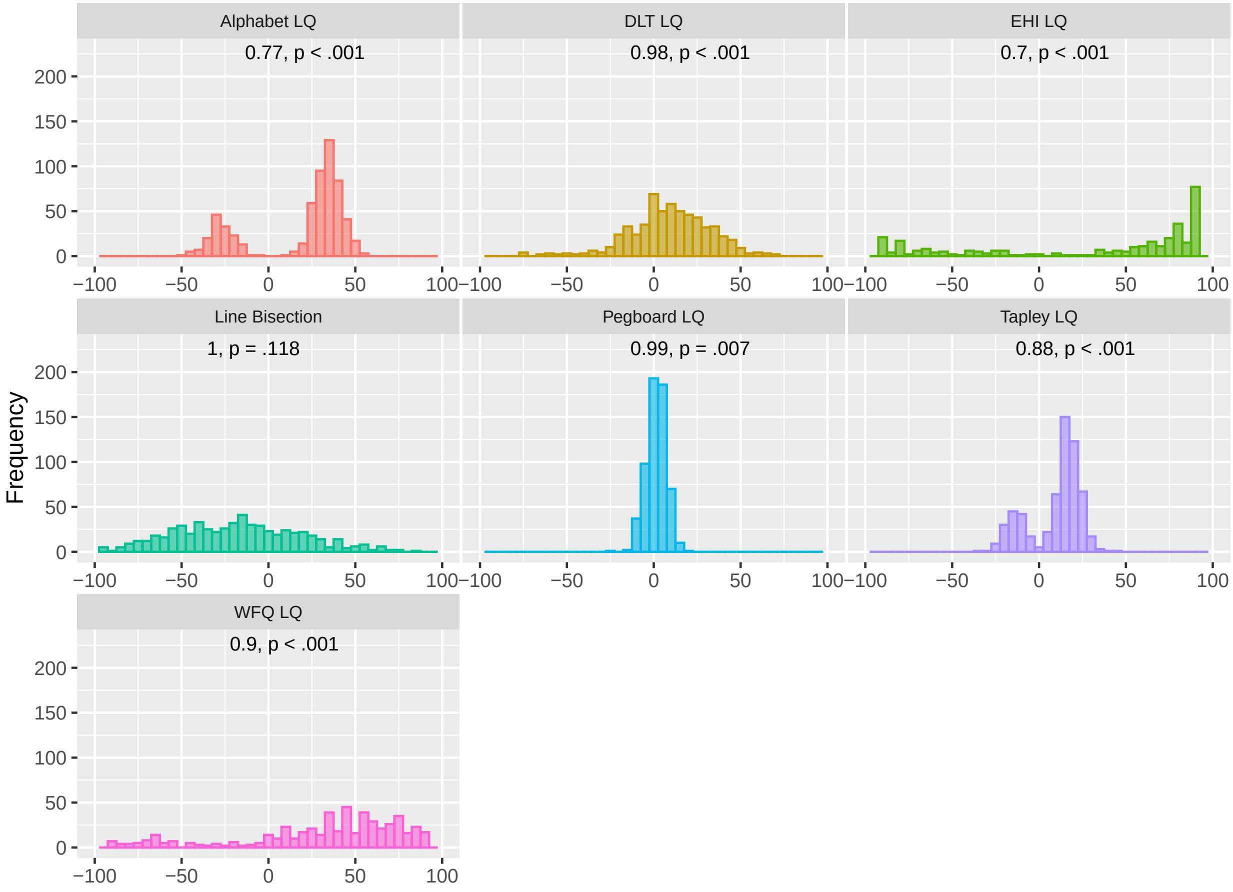

Figure S1

Histograms of asymmetry measures before transformation and results of Shapiro-Wilk test for normality.

The Line Bisection score is the only variable following a normal distribution indicated by the Shapiro-Wilk test. The EHI and WFQ LQs show the expected J-shaped distribution, which is more pronounced for the EHI LQ. The distribution of the Alphabet

LQ and Tapley LQ are clearly bimodal, which is most likely due to the nature of the tasks. The DLT LQ resembles a normal distribution visually, while the Peg LQ is strongly leptocurtic.

Figure S2 shows the distributions of quantitative asymmetry measures after transformation:

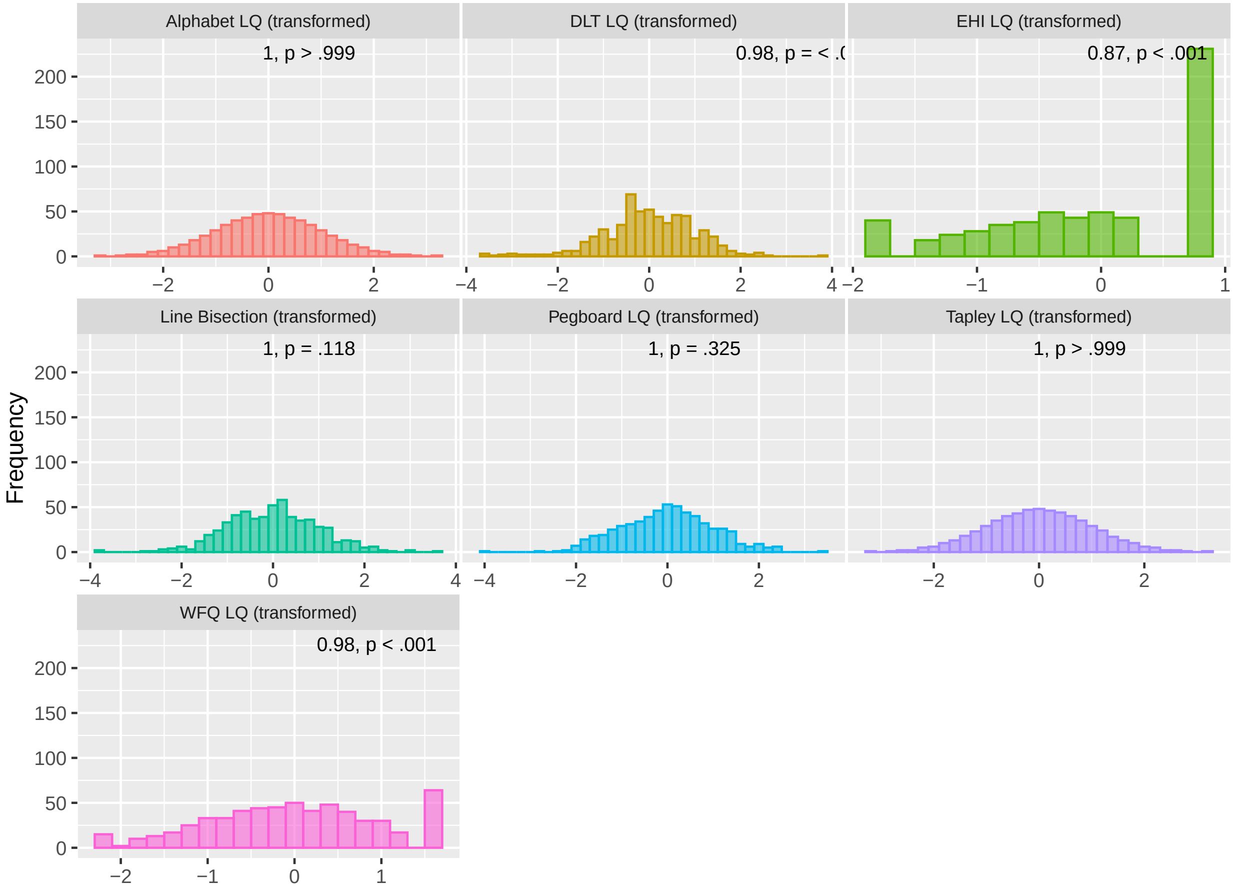

Figure S2
Histograms of asymmetry measures after transformation and results of Shapiro-Wilk test for normality.

The Shapiro-Wilk test indicated normality after transformation for the Alphabet LQ (orderNorm Transformation), the Line Bisection score (center_scale(x) Transformation), the Peg LQ (Standardized Yeo-Johnson Transformation), and the Tapley LQ (orderNorm Transformation). The DLT LQ (Standardized Yeo-Johnson

Transformation) still deviated from normality according to the Shapiro-Wilk test, as did the EHI LQ (orderNorm Transformation) and WFQ LQ (orderNorm Transformation) which still showed the J-shaped distribution.

Transformations of quantitative birth factors

Figure S3 shows the distribution of the six quantitative birth factors before transformation:

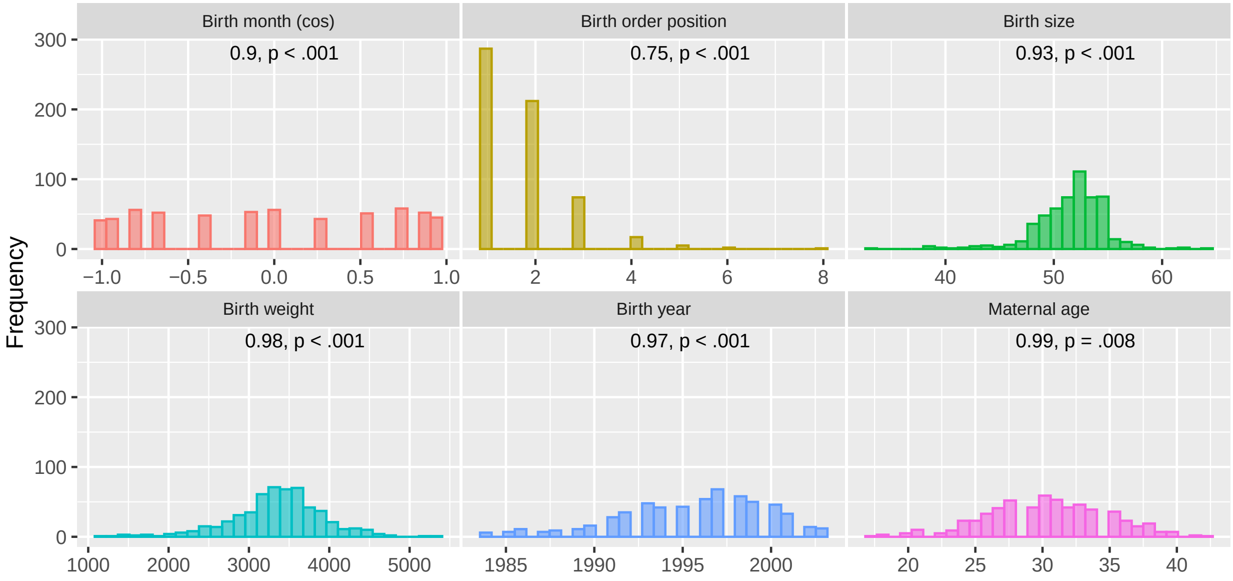

Figure S3
Histograms of quantitative birth factors before transformation and results of Shapiro-Wilk test for normality.

The birth month variable showed a strongly mesokurtic distribution, while birth order position was strongly right-skewed. Birth size was left-skewed, while birth weight, birth year, and maternal age showed no skew, but showed a mesocurtic distribution. None of the measures showed normality according to the Shapiro-Wilk test.

Figure S4 shows the distributions of quantitative birth factors after transformation:

Transformation did not result in normality according to the Shapiro-Wilk test for any of the variables. Birth month (orderNorm Transformation), birth size (Standardized Box Cox Transformation), birth weight (orderNorm Transformation), birth year (orderNorm Transformation), and maternal age (center_scale(x) Transformation) seemed to have improved and were deemed sufficiently normally distributed to be used in the regression models. In contrast, birth order position (Standardized Box Cox

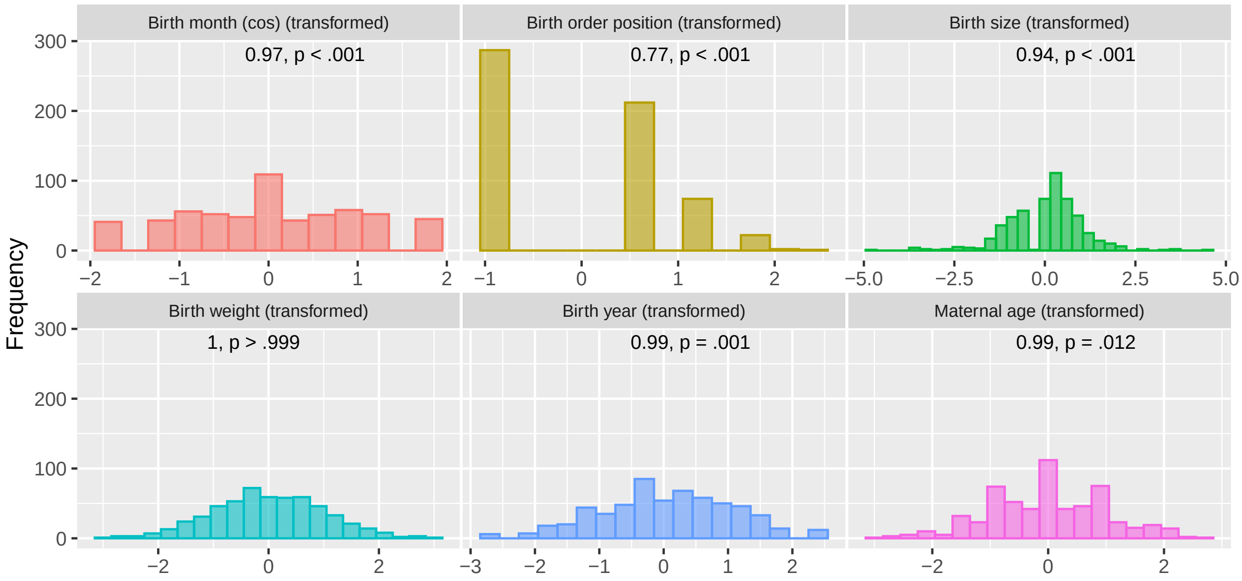

Figure S4

Histograms of quantitative birth factors after transformation and results of Shapiro-Wilk test for normality.

Transformation) still showed a strongly skewed distribution. We therefore decided to dichotomize birth order position (first vs. laterborn), resulting in five quantitative birth factors mentioned in the main manuscript.

Transformations of clinical questionnaires

Figure S5 shows the clinical questionnaires (overall sum scores as well as subscale scores) before transformation:

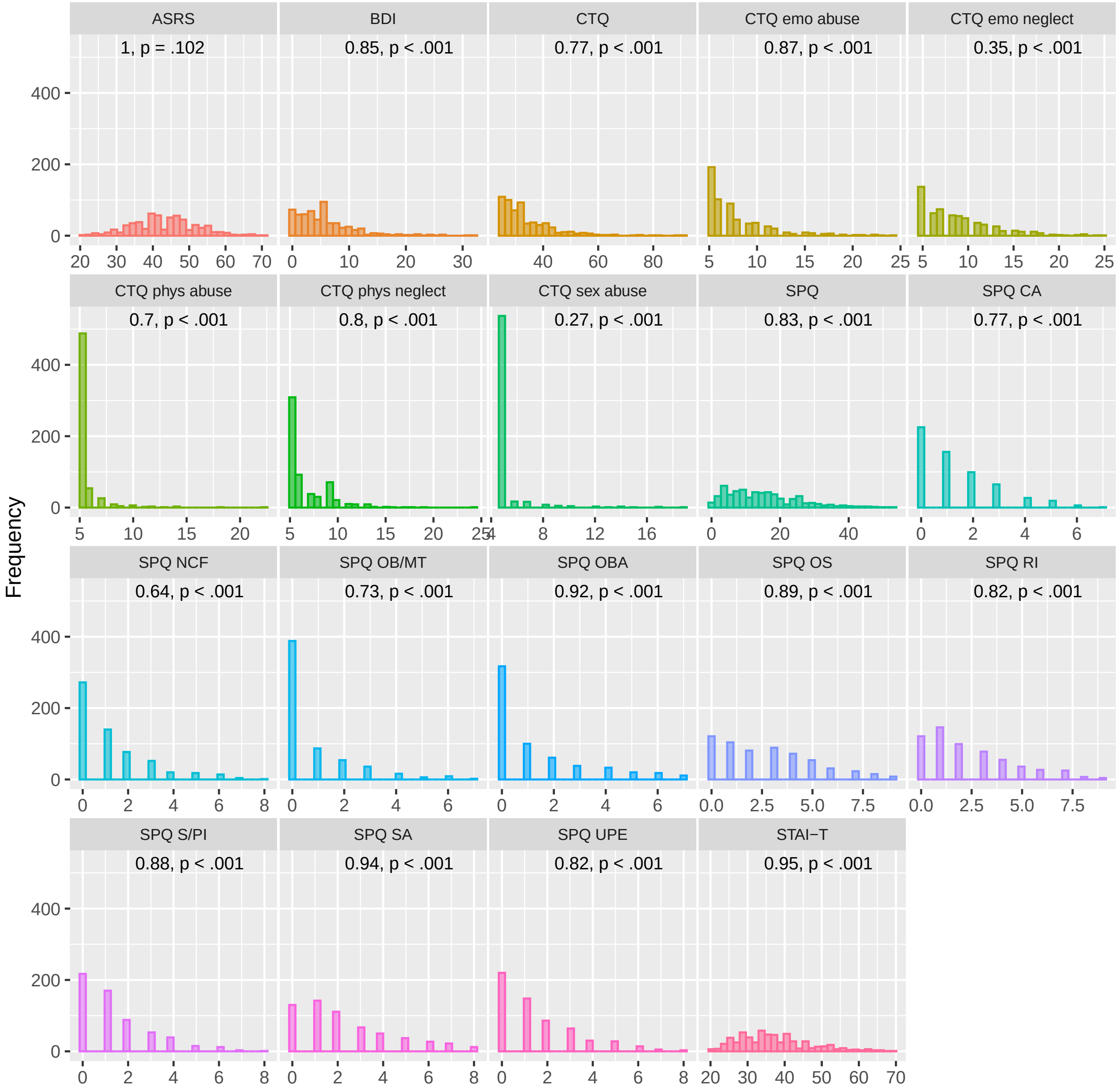

Figure S5

Histograms of clinical questionnaires before transformation and results of Shapiro-Wilk test for normality.

According to the Shapiro-Wilk test, only the ADHD score was normally distributed,

while the BDI score, CTQ score, SPQ score, and STAI-T score as well as subscales were right-skewed.

Figure S6 shows the distributions of clinical questionnaires after transformation:

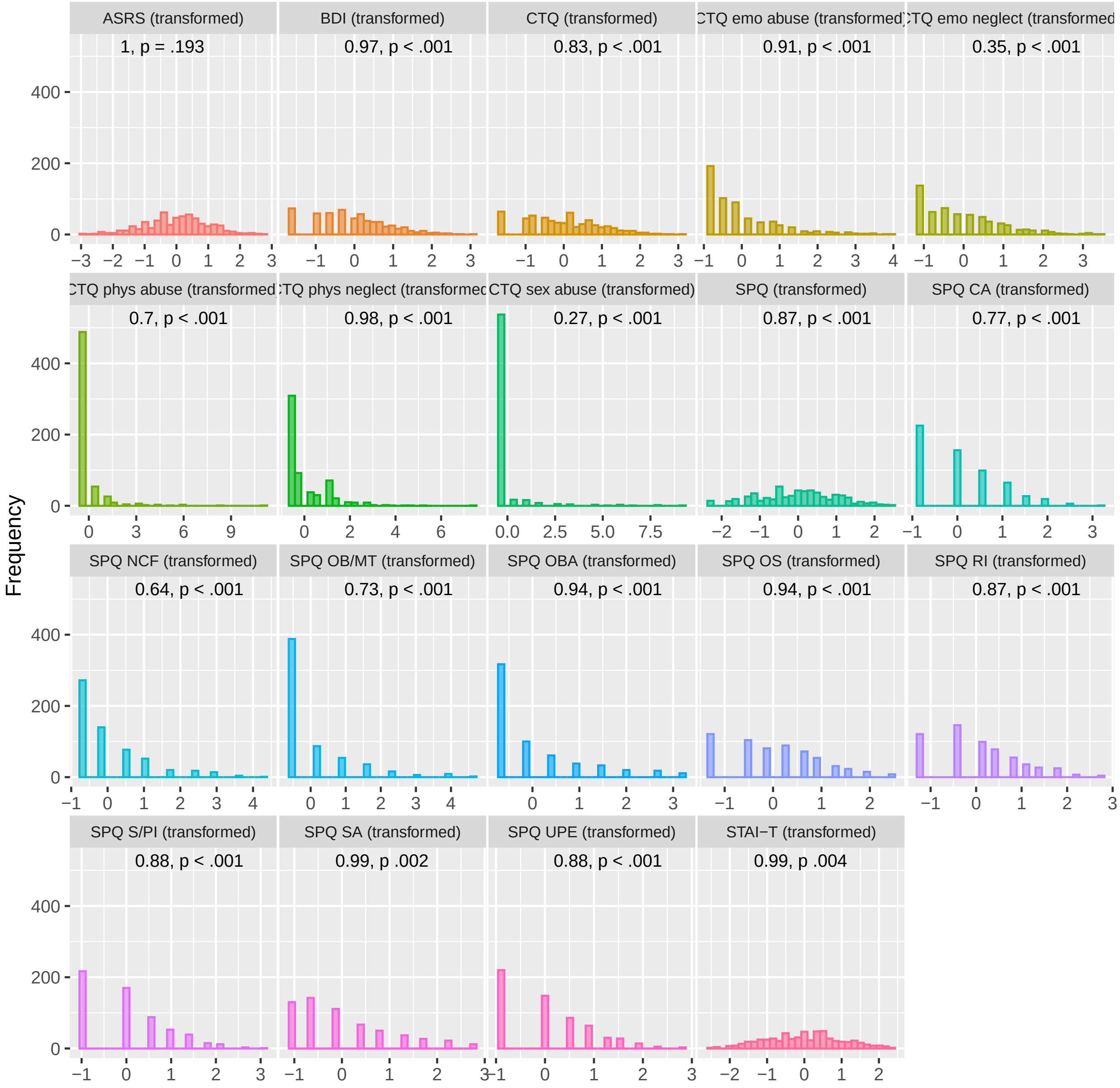

Figure S6

Histograms of clinical questionnaires after transformation and results of Shapiro-Wilk test for normality.

Besides the ADHD score (center_scale(x) Transformation), none of the clinical

questionnaire scores is normally distributed according to the Shapiro-Wilk test. Sum scores, however, seemed to be less skewed after transformation for the BDI score (orderNorm Transformation), the CTQ score (orderNorm Transformation), the SPQ score (Standardized Yeo-Johnson Transformation), and the STAI-T score (Standardized Box Cox Transformation). Based on the strong right-skew of the subscales, we decided to only analyse the overall sum scores rather than the subscales.

Intercorrelations of asymmetry measures

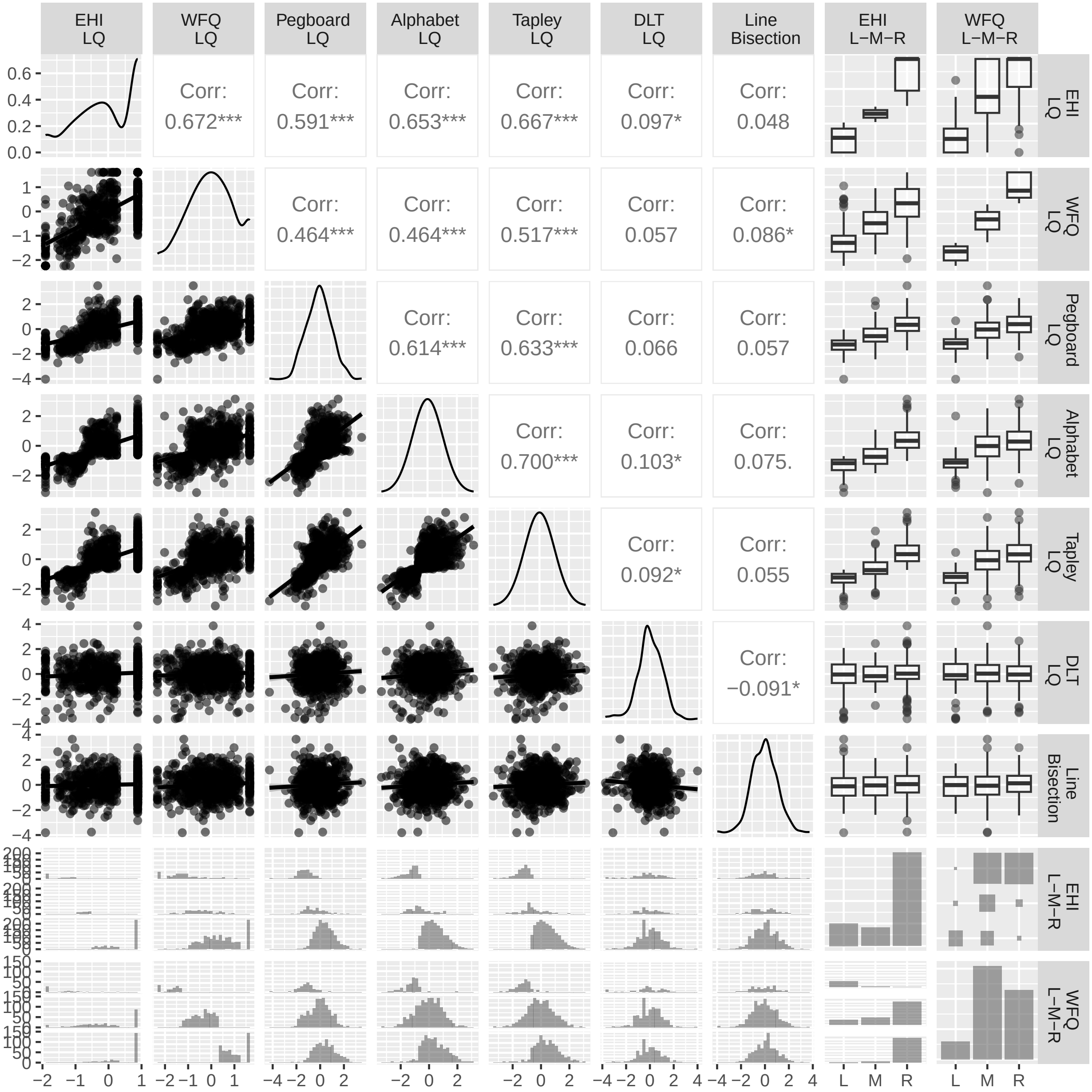

Figure S7

Intercorrelations of asymmetry measures. Cells on the diagonal show distributions of quantitative asymmetry measures and bar plots for categorical asymmetry measures. Cells below the diagonal show scatterplots for quantitative vs. quantitative asymmetry measures (e.g., EHI LQ vs. WFQ LQ), grouped histograms for categorical vs. quantitative asymmetry measures (e.g., EHI LQ vs. EHI L-M-R), and grouped histograms for categorical vs. categorical asymmetry measures (EHI L-M-R vs. WFQ L-M-R). Cells above the diagonal show correlation coefficients for quantitative vs. quantitative asymmetry measures (e.g., WFQ LQ vs. EHI LQ), box plots for categorical vs. quantitative asymmetry measures (e.g., EHI L-M-R vs. EHI LQ), and grouped histograms for categorical vs. categorical asymmetry measures (WFQ L-M-R vs. EHI L-M-R).

Intercorrelations of birth factors

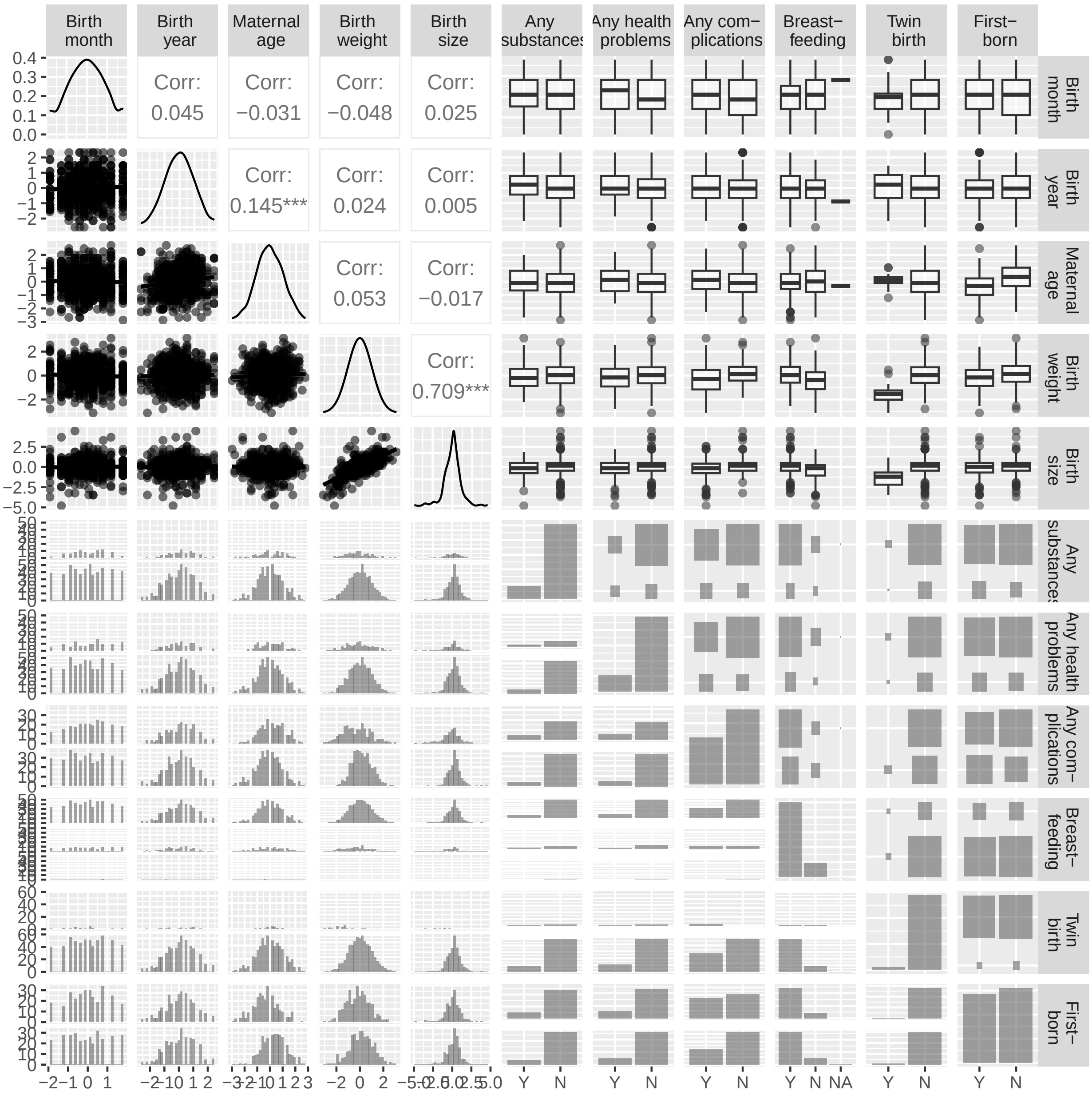

Figure S8

Intercorrelations of birth factors. Cells on the diagonal show distributions of quantitative birth factors and bar plots for categorical birth factors. Cells below the diagonal show scatterplots for quantitative vs. quantitative birth factors (e.g., birth month vs. birth year), grouped histograms for categorical vs. quantitative asymmetry measures (e.g., birth month vs. Any substances), and grouped histograms for categorical vs. categorical asymmetry measures (e.g., Any substances vs. Any health problems). Cells above the diagonal show correlation coefficients for quantitative vs. quantitative asymmetry measures (e.g., birth year vs. birth month), box plots for categorical vs. quantitative asymmetry measures (e.g., Any substances vs. birth month), and grouped histograms for categorical vs. categorical asymmetry measures (e.g., Any health problems vs. Any substances).

Intercorrelations of clinical questionnaires

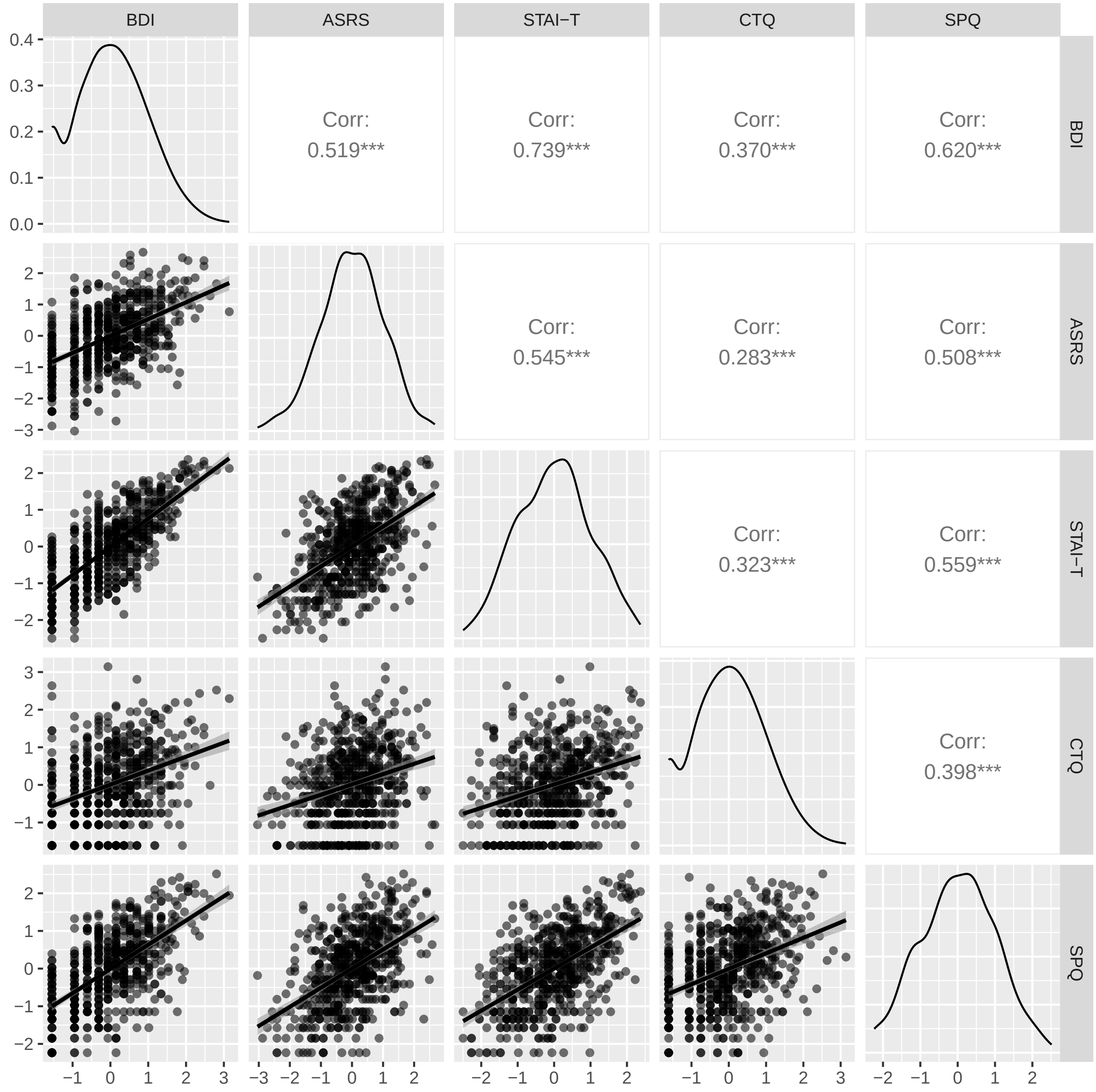

Figure S9

Intercorrelations of clinical questionnaires. Cells on the diagonal show distributions of clinical questionnaires. Cells below the diagonal show scatterplots. Cells above the diagonal show correlation coefficients.

Results

Part 2: Linear regressions: Asymmetry ~ quantitative birth factors

Table S1

Quantitative asymmetry measures as a function of quantitative birth factors (linear regression).

Model	Outcome	Predictor	Intercept	ß	SE	t	p_{raw}	R^2	p_{FDR}*
1	EHI LQ	Birth month	-0.04	-0.02	0.04	-0.46	.646	0.00	.503
2	WFQ LQ	Birth month	-0.01	0.00	0.04	0.03	.979	0.00	.559
3	Pegboard LQ	Birth month	0.00	-0.03	0.04	-0.60	.548	0.00	.498
4	Alphabet LQ	Birth month	0.00	-0.02	0.04	-0.45	.654	0.00	.503
5	Tapley LQ	Birth month	0.00	0.02	0.04	0.47	.638	0.00	.503
6	DLT LQ	Birth month	0.00	0.03	0.04	0.80	.424	0.00	.498
7	Line Bisection	Birth month	0.00	-0.05	0.04	-1.11	.268	0.00	.383
8	EHI LQ	Birth year	-0.04	0.01	0.04	0.35	.726	0.00	.538
9	WFQ LQ	Birth year	-0.01	-0.01	0.04	-0.22	.822	0.00	.543
10	Pegboard LQ	Birth year	0.00	0.05	0.04	1.29	.198	0.00	.383
11	Alphabet LQ	Birth year	0.00	0.03	0.04	0.67	.506	0.00	.498
12	Tapley LQ	Birth year	0.00	0.06	0.04	1.48	.139	0.00	.383
13	DLT LQ	Birth year	0.00	-0.07	0.04	-1.70	.090	0.00	.383
14	Line Bisection	Birth year	0.00	0.05	0.04	1.13	.260	0.00	.383
15	EHI LQ	Maternal age	-0.04	-0.07	0.04	-1.85	.065	0.01	.383
16	WFQ LQ	Maternal age	-0.01	-0.03	0.04	-0.66	.510	0.00	.498
17	Pegboard LQ	Maternal age	0.00	-0.05	0.04	-1.25	.213	0.00	.383
18	Alphabet LQ	Maternal age	0.00	-0.07	0.04	-1.78	.075	0.00	.383
19	Tapley LQ	Maternal age	0.00	-0.07	0.04	-1.73	.085	0.00	.383
20	DLT LQ	Maternal age	0.00	0.02	0.04	0.48	.628	0.00	.503
21	Line Bisection	Maternal age	0.00	0.04	0.04	1.00	.318	0.00	.419
22	EHI LQ	Birth weight	-0.04	0.00	0.04	0.06	.954	0.00	.559
23	WFQ LQ	Birth weight	-0.01	0.00	0.04	-0.10	.917	0.00	.556
24	Pegboard LQ	Birth weight	0.00	0.01	0.04	0.26	.799	0.00	.543
25	Alphabet LQ	Birth weight	-0.01	0.00	0.04	-0.10	.918	0.00	.556
26	Tapley LQ	Birth weight	-0.01	-0.01	0.04	-0.20	.841	0.00	.543
27	DLT LQ	Birth weight	0.03	0.01	0.04	0.20	.842	0.00	.543
28	Line Bisection	Birth weight	-0.03	0.06	0.04	1.50	.134	0.00	.383
29	EHI LQ	Birth size	-0.04	-0.02	0.04	-0.62	.533	0.00	.498
30	WFQ LQ	Birth size	-0.02	-0.05	0.04	-1.32	.188	0.00	.383
31	Pegboard LQ	Birth size	0.00	0.03	0.04	0.68	.498	0.00	.498
32	Alphabet LQ	Birth size	0.00	-0.05	0.04	-1.23	.218	0.00	.383
33	Tapley LQ	Birth size	-0.01	-0.06	0.04	-1.32	.189	0.00	.383
34	DLT LQ	Birth size	0.04	0.04	0.04	0.96	.335	0.00	.419
35	Line Bisection	Birth size	-0.02	0.05	0.04	1.14	.256	0.00	.383

Note. *FDR correction for 20 independent tests.

Part 3: ANOVAs: Asymmetry ~ binary birth factors

Table S2

Quantitative asymmetry measures as a function of binary birth factors (ANOVA).

							No		Yes		
Model	Outcome	Predictor	Levene p	F	p_{raw}	η^2	M	SE	M	SE	p_{FDR}*
1	EHI LQ	Any substances	.225	0.02	.882	0.00	-0.04	0.04	-0.05	0.09	.569
2	WFQ LQ	Any substances	.966	0.11	.743	0.00	-0.02	0.04	0.02	0.10	.569
3	Pegboard LQ	Any substances	.929	0.75	.388	0.00	-0.02	0.04	0.09	0.11	.569
4	Alphabet LQ	Any substances	.852	0.50	.482	0.00	-0.01	0.04	0.07	0.11	.569
5	Tapley LQ	Any substances	.762	0.03	.863	0.00	0.00	0.04	0.02	0.11	.569
6	DLT LQ	Any substances	.933	0.08	.774	0.00	0.00	0.04	0.03	0.11	.569
7	Line Bisection	Any substances	.362	0.16	.690	0.00	-0.01	0.04	0.04	0.11	.569
8	EHI LQ	Any health problems	.873	0.04	.841	0.00	-0.04	0.04	-0.03	0.08	.569
9	WFQ LQ	Any health problems	.668	0.02	.887	0.00	-0.01	0.04	-0.02	0.09	.569
10	Pegboard LQ	Any health problems	.685	0.66	.416	0.00	-0.02	0.04	0.07	0.10	.569
11	Alphabet LQ	Any health problems	.762	0.12	.733	0.00	-0.01	0.04	0.03	0.10	.569
12	Tapley LQ	Any health problems	.643	0.00	.988	0.00	0.00	0.04	0.00	0.10	.588
13	DLT LQ	Any health problems	.633	0.87	.351	0.00	-0.02	0.04	0.08	0.10	.569
14	Line Bisection	Any health problems	.996	0.23	.630	0.00	0.01	0.04	-0.04	0.10	.569
15	EHI LQ	Any birth complications	.345	1.14	.286	0.00	-0.01	0.04	-0.09	0.06	.569

Table S2 continued

							No		Yes		
Model	Outcome	Predictor	Levene p	F	p_{raw}	η^2	M	SE	M	SE	p_{FDR}*
16	WFQ LQ	Any birth complications	.156	0.01	.912	0.00	-0.01	0.05	-0.02	0.06	.570
17	Pegboard LQ	Any birth complications	.938	4.69	.031	0.01	0.07	0.05	-0.11	0.07	.369
18	Alphabet LQ	Any birth complications	.982	3.58	.059	0.01	0.06	0.05	-0.10	0.07	.369
19	Tapley LQ	Any birth complications	.161	1.38	.241	0.00	0.04	0.05	-0.06	0.07	.569
20	DLT LQ	Any birth complications	.177	0.39	.530	0.00	0.02	0.05	-0.03	0.07	.569
21	Line Bisection	Any birth complications	.216	0.00	.953	0.00	0.00	0.05	0.00	0.07	.581
22	EHI LQ	Breastfeeding	.379	0.16	.687	0.00	-0.07	0.09	-0.03	0.04	.569
23	WFQ LQ	Breastfeeding	.418	0.06	.809	0.00	0.01	0.10	-0.02	0.04	.569
24	Pegboard LQ	Breastfeeding	.711	0.39	.535	0.00	-0.06	0.10	0.01	0.04	.569
25	Alphabet LQ	Breastfeeding	.466	0.56	.454	0.00	-0.07	0.10	0.01	0.04	.569
26	Tapley LQ	Breastfeeding	.547	0.10	.754	0.00	-0.03	0.10	0.00	0.04	.569
27	DLT LQ	Breastfeeding	.019	0.83	.363	0.00	0.08	0.10	-0.02	0.04	.569
28	Line Bisection	Breastfeeding	.212	0.57	.449	0.00	-0.07	0.10	0.01	0.04	.569
29	EHI LQ	Twin birth	.990	0.16	.692	0.00	-0.04	0.04	-0.12	0.20	.569
30	WFQ LQ	Twin birth	.491	0.11	.740	0.00	-0.01	0.04	-0.08	0.22	.569

Table S2 continued

							No		Yes		
Model	Outcome	Predictor	Levene p	F	p_{raw}	η^2	M	SE	M	SE	p_{FDR}*
31	Pegboard LQ	Twin birth	.794	0.07	.799	0.00	0.00	0.04	-0.06	0.22	.569
32	Alphabet LQ	Twin birth	.419	0.33	.567	0.00	0.00	0.04	-0.13	0.22	.569
33	Tapley LQ	Twin birth	.395	0.03	.871	0.00	0.00	0.04	0.04	0.22	.569
34	DLT LQ	Twin birth	.243	0.38	.536	0.00	0.00	0.04	0.14	0.22	.569
35	Line Bisection	Twin birth	.441	0.12	.730	0.00	0.00	0.04	0.08	0.22	.569
36	EHI LQ	Firstborn	.106	3.72	.054	0.01	-0.11	0.05	0.03	0.05	.369
37	WFQ LQ	Firstborn	.980	1.44	.230	0.00	-0.06	0.06	0.04	0.06	.569
38	Pegboard LQ	Firstborn	.932	4.73	.030	0.01	-0.08	0.06	0.09	0.06	.369
39	Alphabet LQ	Firstborn	.274	2.58	.109	0.00	-0.06	0.06	0.07	0.06	.454
40	Tapley LQ	Firstborn	.238	3.09	.079	0.00	-0.07	0.06	0.08	0.06	.395
41	DLT LQ	Firstborn	.693	0.30	.583	0.00	0.02	0.06	-0.02	0.06	.569
42	Line Bisection	Firstborn	.961	1.04	.308	0.00	0.04	0.06	-0.04	0.06	.569

Note. *FDR correction for 25 independent tests.

Part 4: ANOVAs: Asymmetry ~ quantitative birth factors

Table S3

Categorical asymmetry measures (L-M-R) as a function of quantitative birth factors.

							Right - Mixed		Right - Left		Mixed - Left		
Model	Outcome	Predictor	Levene p	F	p_{raw}	η^2	Diff	p_{Bonf}	Diff	p_{Bonf}	Diff	p_{Bonf}	p_{FDR}*
1	Birth month	EHI L-M-R	.607	0.07	.933	0.00	-0.02	> .999	0.03	> .999	0.05	> .999	.373
2	Birth month	WFQ L-M-R	.435	0.54	.581	0.00	-0.07	> .999	0.03	> .999	0.10	> .999	.356
3	Birth year	EHI L-M-R	.310	0.93	.394	0.00	0.04	> .999	0.15	.518	0.11	> .999	.356
4	Birth year	WFQ L-M-R	.665	1.30	.273	0.00	-0.13	.380	-0.01	> .999	0.12	> .999	.356
5	Maternal age	EHI L-M-R	.196	4.28	.014	0.01	0.14	.690	-0.27	.049	-0.41	.017	.056
6	Maternal age	WFQ L-M-R	.397	0.27	.767	0.00	-0.02	> .999	-0.10	> .999	-0.09	> .999	.356
7	Birth weight	EHI L-M-R	.305	0.32	.725	0.00	-0.10	> .999	0.00	> .999	0.10	> .999	.356
8	Birth weight	WFQ L-M-R	.026	0.28	.757	0.00	-0.04	> .999	0.05	> .999	0.10	> .999	.356
9	Birth size	EHI L-M-R	.628	0.94	.390	0.00	-0.14	.756	-0.11	> .999	0.03	> .999	.356
10	Birth size	WFQ L-M-R	.065	0.22	.801	0.00	-0.02	> .999	-0.10	> .999	-0.08	> .999	.356

Note. *FDR correction for 4 independent tests.

Part 5: Chi square tests: Asymmetry ~ binary birth factors

Table S4

Categorical asymmetry measures (L-M-R) as a function of binary birth factors.

Outcome	Predictor	χ^2	df	p_{raw}	p_{FDR}*
EHI L-M-R	Any substances	1.48	2.00	.476	.339
WFQ L-M-R	Any substances	1.49	2.00	.476	.339
EHI L-M-R	Any health problems	1.67	2.00	.435	.339
WFQ L-M-R	Any health problems	0.78	2.00	.678	.339
EHI L-M-R	Any birth complications	2.05	2.00	.359	.339
WFQ L-M-R	Any birth complications	1.12	2.00	.573	.339
EHI L-M-R	Breastfeeding	0.17	2.00	.920	.402
WFQ L-M-R	Breastfeeding	0.99	2.00	.610	.339
EHI L-M-R	Twin birth	0.07	2.00	.966	.402
WFQ L-M-R	Twin birth	1.30	2.00	.523	.339
EHI L-M-R	Firstborn	6.21	2.00	.045	.224
WFQ L-M-R	Firstborn	0.84	2.00	.658	.339

Note. *FDR correction for 5 independent tests.

Part 6: Linear regressions: Clinical questionnaires ~ asymmetry

Table S5

Clinical questionnaires (overall scores) as a function of quantitative asymmetry measures (linear regression).

Model	Outcome	Predictor	Intercept	ß	SE	t	p_{raw}	R^2	p_{FDR}*
1	BDI	EHI LQ	0.01	-0.06	0.04	-1.30	.195	0.00	.266
2	ASRS	EHI LQ	0.00	-0.05	0.05	-1.02	.309	0.00	.274
3	STAI-T	EHI LQ	0.00	-0.05	0.05	-1.01	.311	0.00	.274
4	CTQ	EHI LQ	0.01	0.02	0.05	0.47	.638	0.00	.383
5	SPQ	EHI LQ	0.00	-0.10	0.05	-2.22	.027	0.01	.129
6	BDI	WFQ LQ	0.01	-0.06	0.04	-1.50	.133	0.00	.222
7	ASRS	WFQ LQ	0.00	-0.09	0.04	-2.16	.031	0.01	.129
8	STAI-T	WFQ LQ	0.00	-0.03	0.04	-0.67	.502	0.00	.322
9	CTQ	WFQ LQ	0.01	0.00	0.04	0.12	.901	0.00	.386
10	SPQ	WFQ LQ	0.00	-0.07	0.04	-1.67	.095	0.00	.216
11	BDI	Pegboard LQ	0.01	-0.03	0.04	-0.65	.515	0.00	.322
12	ASRS	Pegboard LQ	0.00	-0.01	0.04	-0.17	.865	0.00	.386
13	STAI-T	Pegboard LQ	0.00	-0.03	0.04	-0.83	.408	0.00	.322
14	CTQ	Pegboard LQ	0.01	-0.01	0.04	-0.22	.825	0.00	.386
15	SPQ	Pegboard LQ	0.00	-0.05	0.04	-1.22	.224	0.00	.274
16	BDI	Alphabet LQ	0.01	-0.08	0.04	-2.04	.042	0.01	.129
17	ASRS	Alphabet LQ	0.00	-0.03	0.04	-0.70	.487	0.00	.322
18	STAI-T	Alphabet LQ	0.00	-0.04	0.04	-0.85	.394	0.00	.322
19	CTQ	Alphabet LQ	0.01	0.05	0.04	1.15	.250	0.00	.274
20	SPQ	Alphabet LQ	0.00	-0.11	0.04	-2.73	.006	0.01	.090
21	BDI	Tapley LQ	0.01	-0.05	0.04	-1.35	.176	0.00	.264
22	ASRS	Tapley LQ	0.00	-0.02	0.04	-0.40	.690	0.00	.383
23	STAI-T	Tapley LQ	0.00	-0.04	0.04	-1.08	.278	0.00	.274
24	CTQ	Tapley LQ	0.01	0.02	0.04	0.38	.706	0.00	.383
25	SPQ	Tapley LQ	0.00	-0.07	0.04	-1.64	.101	0.00	.216
26	BDI	DLT LQ	0.01	-0.01	0.04	-0.14	.888	0.00	.386
27	ASRS	DLT LQ	0.00	0.01	0.04	0.14	.887	0.00	.386
28	STAI-T	DLT LQ	0.00	0.01	0.04	0.33	.740	0.00	.383
29	CTQ	DLT LQ	0.01	-0.08	0.04	-2.03	.043	0.01	.129
30	SPQ	DLT LQ	0.00	-0.03	0.04	-0.78	.435	0.00	.322
31	BDI	Line Bisection	0.01	-0.03	0.04	-0.73	.463	0.00	.322
32	ASRS	Line Bisection	0.00	0.00	0.04	0.12	.901	0.00	.386
33	STAI-T	Line Bisection	0.00	-0.04	0.04	-1.05	.295	0.00	.274
34	CTQ	Line Bisection	0.01	-0.06	0.04	-1.55	.122	0.00	.222
35	SPQ	Line Bisection	0.00	-0.01	0.04	-0.34	.732	0.00	.383

Note. *FDR correction for 15 independent tests.

Part 7: ANOVA: Clinical questionnaires ~ asymmetry

Table S6

Clinical questionnaires (overall scores) as a function of categorical asymmetry measures (L-M-R) (ANOVA).

							Right - Mixed		Right - Left		Mixed - Left		
Model	Outcome	Predictor	Levene p	F	p_{raw}	η^2	Diff	p_{Bonf}	Diff	p_{Bonf}	Diff	p_{Bonf}	p_{FDR}*
1	BDI	EHI L-M-R	.277	1.34	.262	0.00	-0.18	.365	-0.08	> .999	0.10	> .999	.087
2	ASRS	EHI L-M-R	.062	1.43	.239	0.00	-0.20	.275	-0.02	> .999	0.18	.665	.087
3	STAI-T	EHI L-M-R	.483	0.73	.482	0.00	-0.14	.723	-0.06	> .999	0.09	> .999	.145
4	CTQ	EHI L-M-R	.188	2.28	.103	0.01	-0.12	.907	0.18	.295	0.30	.115	.052
5	SPQ	EHI L-M-R	.203	3.82	.022	0.01	-0.33	.021	-0.12	.901	0.21	.462	.022
6	BDI	WFQ L-M-R	.036	3.07	.047	0.01	-0.17	.114	0.08	> .999	0.25	.195	.028
7	ASRS	WFQ L-M-R	.015	7.28	.001	0.02	-0.29	.003	0.09	> .999	0.38	.022	.003
8	STAI-T	WFQ L-M-R	.778	3.46	.032	0.01	-0.17	.142	0.14	> .999	0.31	.087	.024
9	CTQ	WFQ L-M-R	.754	1.41	.246	0.00	-0.02	> .999	0.21	.427	0.23	.288	.087
10	SPQ	WFQ L-M-R	.622	4.70	.009	0.02	-0.25	.011	-0.02	> .999	0.23	.302	.013

Note. *FDR correction for 3 independent tests.

References

Peterson, RyanA. (2021). Finding optimal normalizing transformations via bestNormalize. *The R Journal*, *13*(1), 310. https://doi.org/10.32614/RJ-2021-041
